# Protective Effects of *Calligonum comosum* as a Natural Remedy to Counteract Pregabalin‐Induced Toxicity: Insights From Chemical Profiling, In Vivo, and In Silico Analyses

**DOI:** 10.1002/fsn3.70681

**Published:** 2025-07-25

**Authors:** Smail Mehda, Ibtissam Laib, Feriel Diab, Raounek Attia, Yousef Benaissa, Attia Hanane, Khiari Rayhana, Meriem Bellabidi, Huda Alsaeedi, David Croun, Mikhael Bechelany, Ahmed Barhoum

**Affiliations:** ^1^ Faculty of Life and Natural Sciences, Department of Agronomy University of El Oued El Oued Algeria; ^2^ Laboratory of Biodiversity and Biotechnology Applications in Agriculture University of El Oued El Oued Algeria; ^3^ Department of Cellular and Molecular Biology El Oued University El Oued Algeria; ^4^ VPRS Laboratory, Chemistry Department Faculty of Mathematics and Matter Sciences. University of KASDI Merbah Ouargla Algeria; ^5^ Higher School of Saharan Agriculture El Oued Algeria; ^6^ Department of Chemistry College of Science, King Saud University Riyadh Saudi Arabia; ^7^ Institut Européen des Membranes, IEM, UMR‐5635, Univ Montpellier, ENSCM, CNRS Montpellier France; ^8^ Functional Materials Group Gulf University for Science and Technology (GUST) Al‐Abdullah Kuwait; ^9^ NanoStruc Research Group, Chemistry Department Faculty of Science, Helwan University Cairo Egypt; ^10^ Chemical and BioPharmaceutical Sciences, Technological University Dublin, Grangegorman Campus Dublin Ireland

**Keywords:** *Calligonum comosum*, hepatoprotection, molecular docking, nephroprotection, pregabalin toxicity, reproductive toxicity

## Abstract

This study explores the therapeutic potential of *Calligonum comosum* extract in alleviating pregabalin (PGB)‐induced toxicity in male Wistar rats, with a focus on hepatic, renal, and reproductive health. PGB exposure led to significant biochemical disturbances, including elevated liver enzymes (AST, ALT, LDH), impaired kidney markers (urea, creatinine, uric acid), reduced reproductive hormones (testosterone, FSH, LH), and notable histopathological damage in liver, kidney, and testicular tissues. Treatment with *C. comosum* extract effectively restored liver and kidney functions and partially corrected hormonal imbalances. The extract reduced AST, ALT, and LDH levels by 18.5%, 25.2%, and 13.7%, respectively. Similarly, urea, creatinine, and uric acid decreased by 30.3%, 38.0%, and 15.2%. Testosterone and LH levels improved, suggesting enhanced reproductive recovery. Histological analyses confirmed reduced inflammation, necrosis, and congestion in treated tissues. Supporting these findings, in silico docking studies showed strong interactions between *C. comosum* phytochemicals and molecular targets linked to toxicity pathways. Quercetin demonstrated the strongest binding (−8.1 to −9.2 kcal/mol), particularly with LXR‐α and GLUT‐1. Rutin showed the highest affinity for GnRH1‐R (−10.4 kcal/mol), while caffeic acid, gallic acid, and chlorogenic acid also exhibited strong interactions, especially with β2 AR (−8.9 kcal/mol). In contrast, PGB displayed weaker binding (−6.0 kcal/mol). These results highlight the protective effects of *C. comosum* and support its potential as a natural remedy for mitigating PGB‐induced hepatorenal and reproductive toxicity.

## Graphical abstract

This study highlights the significant therapeutic effects of *C. comosum* extract in counteracting PGB‐induced toxicity in the liver, kidneys, and testes. Furthermore, in silico analysis offered insights into the protective molecular mechanisms of *C. comosum* phytochemicals against PGB‐induced toxicity. The integration of these computational and experimental findings underscores the potential of *C. comosum* as a natural remedy for PGB‐induced toxicity, providing a strong basis for future studies on natural therapeutic alternatives to mitigate drug‐related adverse effects.

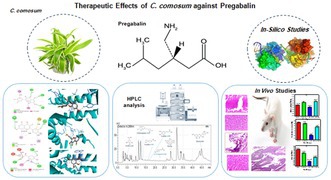

## 1 Introduction

Drugs are essential for treating various diseases. Advances in biotechnology have refined the development of drugs, but understanding their risks (including interactions, side effects, and misuse) is crucial (DiMasi et al. 2003; Olsen and Whalen 2009). Balancing efficacy and safety is a significant challenge, especially in the case of long‐term therapies for chronic conditions (Blackstone and Joseph 2013). For instance, pregabalin (PGB), a highly potent anticonvulsant, has become a cornerstone in the management of epilepsy, neuropathic pain, fibromyalgia, and generalized anxiety disorder. Its therapeutic efficacy is attributed to its ability to bind to voltage‐gated calcium channels, rendering it six times more potent than gabapentin and providing substantial relief for patients facing challenging conditions (Loftus and Wright 2014), (Bockbrader et al. 2010; Martinotti et al. 2013). However, the darker side of PGB emerges through its potential for misuse and dependence, particularly among younger adults. What starts as a legitimate treatment can quickly devolve into a dangerous pattern of abuse, as the drug's euphoric effects make it an attractive target for recreational use. Alarmingly, some individuals have been reported to consume doses up to 20 times the recommended maximum of 900 mg/day, substituting PGB for substances like opium and tramadol, leading to severe outcomes, including cognitive impairment, respiratory depression, and even fatal overdoses (Zaccara et al. 2011).

Misuse of PGB can lead to serious health complications, particularly affecting the liver, kidneys, and reproductive system. The novelty of this study lies in being the first to evaluate the protective effects of *C. comosum* extract against PGB‐induced toxicity in Wistar rats, utilizing a combined in vivo and in silico approach. Mechanistically, PGB triggers hepatotoxicity through oxidative stress and mitochondrial dysfunction, leading to excessive production of reactive oxygen species (ROS), lipid peroxidation, and elevated liver enzymes (ALT, AST). Additionally, PGB disrupts the liver's detoxification capacity by reducing the activity of antioxidant enzymes, further aggravating hepatic damage (Shokry et al. 2020). In addition, PGB‐induced nephrotoxicity manifests as structural damage to renal tubules and glomeruli, resulting in declining renal function, as evidenced by elevated serum creatinine and blood urea nitrogen (BUN) levels. Prolonged exposure to the drug exacerbates oxidative stress, further compromising kidney health, particularly in patients with pre‐existing conditions (Ismail et al. 2022; Shokry et al. 2020). Equally concerning are the effects of PGB on male reproductive health. Studies have shown that misuse of the drug can lead to testicular atrophy, reduced sperm count, and impaired spermatogenesis, primarily driven by increased reactive oxygen species (ROS) and hormonal imbalances, including decreased testosterone levels (Ebrahem et al. 2022; Salah et al. 2024). These indicate the urgent need for vigilant monitoring of liver, kidney, and reproductive health during prolonged or high‐dose PGB use.

As part of ongoing efforts to mitigate the adverse effects of PGB, there is increasing interest in natural therapeutic alternatives with fewer side effects (Cai et al. 2012). Medicinal plants like *C. comosum* (arta) have gained attention for their broad medicinal properties and minimal toxicity (Gasmi et al. 2022), (Smith et al. 2023). Traditionally used for anti‐inflammatory, analgesic, and anxiolytic purposes similar to PGB, *C. comosum* has shown potent therapeutic potential (Smith et al. 2023). For example, Abdallah et al. (2014) (Abdallah et al. 2014) reported its strong antidiabetic effects in animal models, while AI Naqbi (2017) (AI Naqbi 2017) highlighted its antioxidant capacity to combat oxidative stress and prevent chronic diseases. Althurwi et al. (2023) (Althurwi et al. 2023) demonstrated its anti‐inflammatory efficacy, and Mohammed et al. (2018) (Mohammed et al. 2018) confirmed its antimicrobial action against bacterial and fungal pathogens. Additionally, Abdo et al. (2015) and Rzhepakovsky et al. (2022) (Abdo et al. 2015; Rzhepakovsky et al. 2022) showed hepatoprotective effects in liver injury models. Despite these promising findings, no prior studies have examined its protective effects against PGB‐induced hepatic, renal, or reproductive toxicity. Given the central roles of oxidative stress and inflammation in PGB‐mediated organ damage, and the phytochemical richness of *C. comosum* , including flavonoids, tannins, and polyphenols.

This study aimed to evaluate the impact of PGB on liver, kidney, and testicular functions and to assess whether *C. comosum* extract could mitigate PGB‐induced toxicity using in vivo experiments with Wistar rats and in silico molecular docking. Rats were divided into four groups: Control, *C. comosum* extract alone, PGB‐treated, and PGB followed by *C. comosum* extract. The study measured liver function markers (ALT, AST, LDH, and total protein), kidney function markers (urea, creatinine, and uric acid), and testicular function markers (testosterone, FSH, and LH). Additionally, hematological parameters such as RBC, WBC, Hb, Hct, and platelet count, along with biochemical markers including glucose, cholesterol, triglycerides, and albumin, were analyzed. Histopathological examinations were conducted on the liver, kidneys, and testes to evaluate tissue damage, while molecular docking simulations explored the interactions between *C. comosum* phytochemicals and PGB toxicity‐related targets, offering insights into possible protective mechanisms. This study, using a combined in vivo and in silico approach, offers the first comprehensive evaluation of *C. comosum'*s multiorgan protective efficacy against PGB‐induced toxicity. Molecular docking revealed strong interactions between key phytochemicals and proteins involved in oxidative stress, inflammation, and apoptosis. These findings introduce a novel phytotherapeutic strategy, expanding the understanding of PGB's adverse effects and positioning *C. comosum* as a promising natural remedy to reduce organ damage, paving the way for safer alternatives in long‐term PGB therapy.

## 2 Materials and Methods

### 2.1 Collection, Extract and Phytochemical Screening

The aerial parts of *C. comosum* were harvested in March 2023 from El Ghenami, located in the Ouargla province of Algeria (coordinates: 31°57′07″ N, 5°20′00″E), and their identity was confirmed by a botany expert. A voucher specimen was deposited under the number PS/Ca. co 2014 at the Herbarium of the Biology Department in the University of Hamma Lakhdar, El Oued, Algeria. The collected samples were air‐dried and preserved at room temperature (25°C ± 2°C) in a cool, dry, and dark environment until further use. For the extraction process, 10 g of the dried aerial parts of *Calligonum comosum* were immersed in 100 mL of distilled water and kept in the dark at ambient temperature (approximately 25°C) for 24 h. The choice of aqueous extraction was based on its traditional relevance, non‐toxic nature, and suitability for in vivo applications. Water is a safe and biocompatible solvent that facilitates the oral administration of plant extracts in animal studies. After soaking, the mixture was filtered using Whatman No. 1 filter paper, and the filtrate was lyophilized to obtain the dry aqueous extract. All extraction procedures were performed in triplicate under identical conditions to ensure reproducibility and reliability of the phytochemical composition. This step aimed to prevent degradation of light‐sensitive compounds and preserve the plant's antioxidant and pharmacological properties. After the maceration period, the mixture was filtered, and the resulting liquid extract was evaporated at 40°C to obtain the final crude extract. The final extract was weighed, and the yield percentage was calculated using the formula:

$$\text{Yield}\;\left(\%\right)=\left(\text{Weight of}\;\mathrm{dry}\;\text{extract}/\text{Weight of dried plant material}\right)\times 100$$

The dried extract was subsequently weighed and stored at 4°C until further analysis (Tlili, Laib, Salemi, et al. 2024). The *C. comosum* aqueous extract was subjected to a series of standard qualitative phytochemical tests to identify the presence of major bioactive constituents, including alkaloids, flavonoids, tannins, saponins, phenolics, and terpenoids. These tests were conducted in triplicate to ensure reproducibility. The methodologies followed were based on well‐established phytochemical protocols. To validate the specificity of the observed reactions, appropriate positive controls (e.g., quercetin for flavonoids, tannic acid for tannins) and negative controls (reagent blanks and distilled water) were used for each assay. The presence of alkaloids was confirmed using Mayer's (Merck, Germany; analytical grade, ≥ 98%) and Wagner's (Sigma‐Aldrich, USA; analytical grade, ≥ 98) reagents, which produced characteristic cream‐colored and brown to reddish‐brown precipitates, respectively (Barbouchi et al. 2024). Polyphenols were detected by the appearance of a bluish‐black coloration upon the addition of 5% ferric chloride (FeCl_3_, Sigma‐Aldrich (USA), ≥ 98%) solution, indicative of complex formation (Dejene et al. 2025). Flavonoids were identified by the addition of dilute ammonia (NH_4_OH, Merck (Germany), 99%) followed by concentrated sulfuric acid (H_2_SO_4_, Sigma‐Aldrich (USA, 98%)), resulting in a yellow coloration that faded upon standing, confirming their presence (Sarikahya et al. 2019). Tannins were detected by adding ferric chloride (FeCl_3_, Sigma‐Aldrich (USA), 98%), where catechic tannins produced a blue‐black color and gallic tannins yielded a greenish‐black color (İzol et al. 2025). Terpenes and sterols were identified using the Liebermann‐Burchard reagent, producing a distinct blue‐green coloration (Shakya and Das 2025). The presence of saponins was confirmed through the formation of stable froth upon vigorous shaking of the extract with distilled water, a hallmark characteristic of saponins (Nagori et al. 2025). All screening tests were conducted in triplicate under identical experimental conditions.

### 2.2 Phytochemical Analysis

The total phenolic content of the *C. comosum* extract was evaluated using the Folin–Ciocalteu assay. In this method, 1 mL of a 10 wt% Folin–Ciocalteu reagent was mixed with 0.2 mL of the extract. The mixture was left undisturbed for 4 min, after which 800 μL of a saturated sodium carbonate (Na_2_CO_3_, Sigma‐Aldrich (USA), 99%) solution was added. The solution was then incubated at room temperature for 2 h. Absorbance was recorded at 765 nm, and the phenolic content was expressed as milligrams of gallic acid equivalents (mg GAE/g) per gram of extract (Baba and Malik 2015).

The aluminum chloride (AlCl_3_, Sigma‐Aldrich (USA), 99.9%) colorimetric assay was used to determine the total flavonoid content. For this analysis, 1 mL of the extract was combined with 1 mL of a 10 wt% AlCl_3_ solution and left to react at room temperature for 30 min. The absorbance was recorded at 430 nm, and the flavonoid content was calculated as milligrams of quercetin equivalents (mg QE/g) per gram of extract (Shraim et al. 2021).

### 2.3 HPLC Analysis

A detailed analysis of the aqueous extract of *C. comosum* was carried out using a Shimadzu LC20 AL HPLC system equipped with a UV–Vis detector (SPD 20A) and a Shim‐pack VP‐ODS C18 column (4.6 mm × 250 mm, 5 μm particle size). Reverse‐phase chromatography was employed to separate the compounds, using a mobile phase comprising acetonitrile and 0.1% v/v acetic acid (HPLC grades). Both solvents were filtered through 0.22 μm membrane filters and degassed by sonication prior to use to ensure purity and prevent blockages. The gradient elution program was as follows: starting with 10% acetonitrile and 90% acetic acid solution at 0 min, gradually increasing to 50% acetonitrile at 10 min, then to 80% at 20 min, held for 5 min, followed by re‐equilibration to initial conditions. This gradient protocol ensured optimal resolution and peak separation of phytochemical constituents. The flow rate was maintained at 1 mL/min, and the column temperature was held constant at 30°C. Each run was preceded by a 10‐min equilibration phase to stabilize the system. Phenolic compounds were detected at 268 nm, a wavelength selected based on preliminary UV–Vis scans of the extract's phenolics to maximize detection sensitivity across compounds with varying absorbance maxima. Identification was based on retention times compared to authentic standards, and co‐elution was ruled out by confirming peak purity via diode‐array detector spectral analysis across each peak. Concentrations were determined and reported as micrograms per gram of dry extract (μg/g extract).

Each sample was analyzed in triplicate (*n* = 3) to ensure reproducibility. The variability between runs was minimal, with retention time deviations of less than ±0.2 min and relative standard deviation (RSD) values for peak areas below 5%, confirming the precision and consistency of the analysis.

### 2.4 Animal Procurement and Housing

A total of 32 adult male Wistar albino rats (8–10 weeks old), weighing 196.65 ± 3.21 g, were obtained from the Animal Facility of the Pasteur Institute in Algiers, Algeria. Upon delivery to the Department of Molecular and Cellular Biology at the University of El‐Oued, Algeria, the rats were allowed a two‐week acclimatization period under laboratory‐controlled conditions. The environment was maintained at a temperature of 19°C, a relative humidity of 64%, and a 12‐h light/dark cycle. During the study, the animals had ad libitum access to tap water and a standard rat chow diet.

Only adult male Wistar albino rats were used to avoid variability from the female estrous cycle, which affects hormonal, metabolic, and toxicological responses. Male reproductive markers were specifically evaluated to assess testicular dysfunction and recovery. The tests are highly vulnerable to oxidative stress and toxicants like PGB. This approach follows standard toxicological protocols to enhance consistency and sensitivity.

All animal‐related procedures adhered to the ethical guidelines approved by the Institutional Animal Ethical Committee (IAEC) of the University of El‐Oued, Algeria (Approval No. 10/S.C./FL/NS/EU/2024). The study is registered under the reference number 31/2023.

### 2.5 Sub‐Acute Toxicity

The sub‐acute toxicity study was conducted following the general principles of OECD Guideline 407 (Repeated Dose 28‐Day Oral Toxicity Study in Rodents, 2008), with slight modifications tailored to the specific aims of this preliminary investigation. Specifically, a 14‐day exposure period was used as an initial safety assessment. Twenty healthy male Wistar albino rats were randomly assigned into four groups (*n* = 5 per group):

- Group 1: a control group receiving distilled water (0 mg/kg)
- Group 2: Administered *Calligonum comosum* aqueous extract at doses of 100 mg/kg
- Group 3: Administered *Calligonum comosum* aqueous extract at doses of 1000 mg/kg
- Group 4: Administered *Calligonum comosum* aqueous extract at doses of 2000 mg/kg

All groups were administered their respective treatments via oral gavage for 14 consecutive days (Shabana et al. 2023). The selected doses were based on preliminary acute toxicity testing following OECD Guideline 407, which showed no mortality or severe toxicity up to 2000 mg/kg. Furthermore, these doses align with previous reports on the oral safety of *C. comosum* extracts (Saad et al. 2006).

The study aimed to evaluate the preliminary safety profile of the extract through daily non‐invasive monitoring of clinical signs, including body weight changes, mortality, side effects, movement patterns, diarrhea, ocular abnormalities, and death. All observations were systematically recorded and are presented in Table 5, which confirms the absence of adverse effects across all dose levels.

### 2.6 Pregabalin Toxicity and *C. comosum* Effects

After the acclimatization period, the rats were randomly divided into four experimental groups. The dosages of PGB and *C. comosum* were determined based on prior studies and the median lethal dose (LD50 = 3000 mg/kg) of PGB (C_8_H_17_NO_2_, Pfizer (USA), 99.5%), ensuring appropriate exposure levels and treatment duration (Sabry 2012; Shokry et al. 2020). The groups were as follows:

- Group 1 (Control): Rats received no treatment throughout the study duration.
- Group 2 ( *C. comosum* ): Administered *C. comosum* aqueous extract at a dose of 100 mg/kg body weight per day via oral gavage for 35 days.
- Group 3 (PGB): Given PGB at a dose of 200 mg/kg body weight per day, dissolved in drinking water for 35 days.
- Group 4 (PGB + *C. comosum* ): Treated with PGB (200 mg/kg body weight per day) for 35 days, along with *C. comosum* aqueous extract (100 mg/kg body weight per day) administered via oral gavage during the final 15 days of the study.

Different treatment windows were strategically employed to address two key objectives: (i) assessing the long‐term safety profile of *C. comosum* when administered alone over an extended period (Group 2), and (ii) evaluating its therapeutic efficacy when administered after the onset of PGB‐induced toxicity (Group 4). This design mirrors real‐world clinical scenarios, where natural therapeutic agents are typically introduced only after adverse drug reactions are identified. To ensure accurate and consistent PGB exposure, the concentration of PGB in drinking water was carefully adjusted based on the average daily water intake and body weight of each group. Body weights were recorded every 3 days to recalibrate dosing, maintaining an approximate target of 200 mg/kg/day per group. While individual fluid intake could not be monitored due to group housing, this dosing strategy is well‐established and widely accepted in subchronic toxicity studies (Parasuraman 2011). To minimize stress and ensure animal welfare, all procedures including dosing, cage maintenance, and sample collection were performed under standardized conditions and in accordance with institutional ethical guidelines. Daily clinical observations and consistent weight monitoring were conducted to detect any signs of toxicity or distress early. This careful experimental design ensured both the reliability of the data collected and the humane treatment of the animals, thereby reinforcing the scientific and ethical integrity of the study.

At the end of the experiment, each rat was administered ketamine and xylazine to achieve deep sedation over 30 min, ensuring a humane approach. Once fully anesthetized, euthanasia was performed by decapitation using sharp, sterilized blades to collect blood samples directly from the severed neck and to proceed with dissection for liver, kidney, and testicle extraction. Blood samples were collected into centrifuge tubes and left undisturbed to clot. The samples were then centrifuged at 3000 rpm for 15 min to separate the serum, which was carefully extracted and stored at −20°C for subsequent analysis.

#### 2.6.1 Organ Weight, Full Blood Count, and Biochemical Markers

On day 35, the rats were euthanized, and necropsies were performed to check for visible changes in the liver, kidneys, and testes. The organs were subsequently weighed, and their relative weights were calculated as a percentage of the final body weight using the following formula (Shahzad et al. 2012):

$$\text{Relative organ weight}\;\left(\%\right)=\frac{\text{Organ weight}\;\left(g\right)\;}{\text{Final body Weight}\;\left(g\right)\;}\times 100$$

Body weight was tracked regularly throughout the study, with changes expressed as a percentage relative to baseline values. Additionally, blood parameters, including red blood cell (RBC) count, hemoglobin (Hb) concentration, hematocrit (HCT), platelet count, and white blood cell (WBC) count, were measured using a Medonic Automatic Hematology Analyzer (Medonic, Boule Diagnostics AB). Serum glucose and lipid levels were measured using commercial SPINREACT kits (SPINREACT, Girona, Spain). Renal function was evaluated by measuring serum levels of urea, uric acid, and creatinine. Liver function was assessed by determining serum levels of alanine transaminase (ALT), aspartate transaminase (AST), and lactate dehydrogenase (LDH), using SPINREACT kits (SPINREACT, Girona, Spain). Testis function was examined by measuring testosterone using ELISA kits (Thermo Fisher Scientific, Waltham, MA, USA; Abcam, Cambridge, UK). To ensure reliability and reduce variability, all biochemical and ELISA measurements were performed in triplicate for each sample (*n* = 3 technical replicates).

### 2.7 Histological Analysis

**FIGURE 1:**
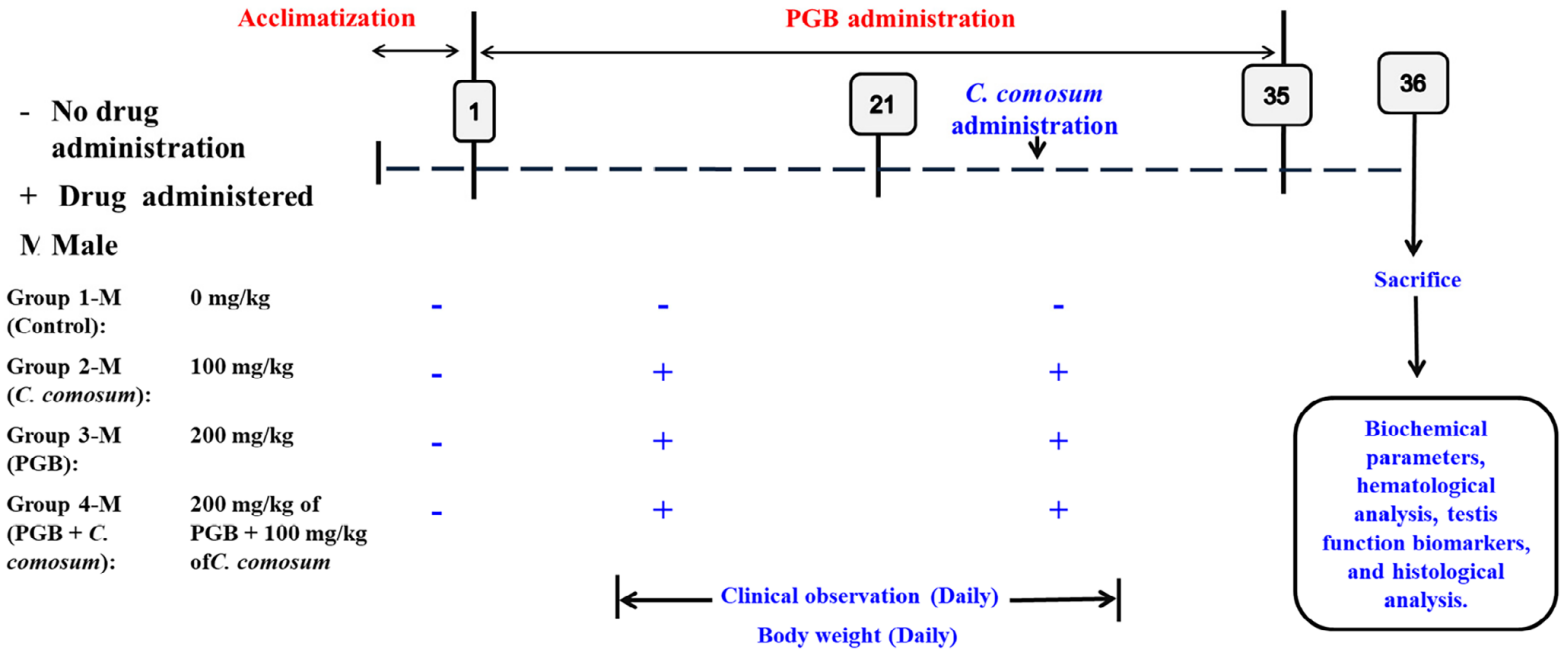
Experimental design diagram for the 35‐day study. Treatments were administered daily following OECD Guideline 407, with modifications tailored to the study's therapeutic evaluation focus.

Liver, kidney, and testicular tissues were collected, grossly examined for visible pathological changes (e.g., discoloration, surface irregularities, vascular congestion, lesions), and preserved in 10% neutral buffered formalin. Although no standardized scoring system was used for gross observations, these findings informed subsequent microscopic assessments. Tissues were processed, paraffin‐embedded, sectioned, and stained with hematoxylin and eosin (H&E) for histological evaluation under a light microscope. A semi‐quantitative scoring system was used to assess the severity of histopathological alterations, including inflammatory infiltration, necrosis, sinusoidal congestion, glomerular destruction, and testicular degeneration (Figure 1). All microscopic evaluations were conducted in a blinded manner by experienced histopathologists to ensure accuracy and minimize bias.

### 2.8 Molecular Docking

Molecular docking was carried out to explore the binding interactions between selected *C. comosum* phytochemicals, PGB, and key target proteins implicated in diverse biological functions. The target proteins included β2 adrenergic receptor (β2 AR; PDB ID: 7XKA, 2.15 Å) (Xu et al. 2023), dipeptidyl peptidase IV (DPP‐IV; 3W2T, 1.90 Å) (Nabeno et al. 2013), glucose transporter 1 (GLUT‐1; 5EQG, 2.30 Å) (Kapoor et al. 2016), liver X receptor alpha (LXR‐α; 1UHL, 2.30 Å), and gonadotropin‐releasing hormone 1 receptor (GnRH1‐R; 7BR3, 2.84 Å) (Yan et al. 2020), all retrieved from the RCSB Protein Data Bank (https://www.rcsb.org/). All were retrieved from the RCSB Protein Data Bank (Berman et al. 2000; Svensson et al. 2003).

The 3D structures of ligands—quercetin (CID: 5280343), rutin (CID: 5280805), chlorogenic acid (CID: 1794427), gallic acid (CID: 370), caffeic acid (CID: 689043), and pregabalin (CID: 5486971)—were obtained from PubChem (https://pubchem.ncbi.nlm.nih.gov/). Protein active sites were identified based on their co‐crystallized ligands and validated using Discovery Studio 21.1's binding site tools.

Molecular docking simulations were performed using AutoDock Vina (Trott and Olson 2010), with grid boxes centered on the pre‐defined active sites to ensure appropriate ligand accommodation. Each ligand–protein complex was subjected to ten genetic algorithm (GA) runs to enhance conformational sampling and ensure reproducibility. The best‐ranked binding affinities (expressed in kcal/mol) were recorded for each ligand. The resulting ligand–receptor interactions were analyzed and visualized using BIOVIA Discovery Studio Visualizer 21.1 (BIOVIA, D. S. 2021), focusing on key parameters such as hydrogen bonding, hydrophobic contacts, and overall binding profiles (BIOVIA, D. S. 2021).

### 2.9 Statistical Analysis

Data are shown as mean ± standard deviation (SD) with five biological replicates per group (*n* = 5). All biochemical and hormonal assays were done in triplicate, and their average was used for analysis. Data normality was checked with the Shapiro–Wilk test, and variance homogeneity with Levene's test. For more than two groups, one‐way ANOVA followed by Tukey's post hoc test was used. For two groups, Student's *t*‐test was applied. Statistical analysis was done using Minitab (Version 13Fr) and Microsoft Excel (Version 2007). A *p* value < 0.05 was considered significant.

## 3 Results and Discussion

### 3.1 Qualitative and Quantitative Analysis of the *C. comosum* Extract

**FIGURE 2:**
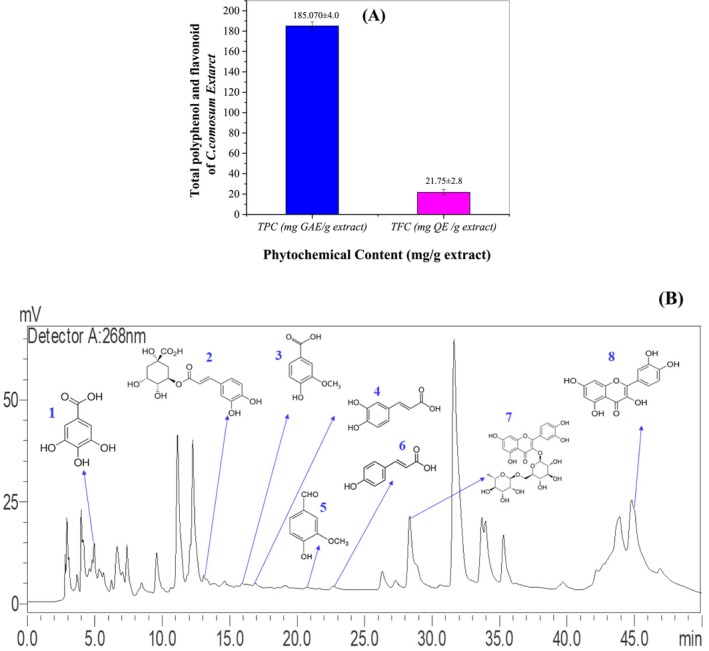
Quantitative and chromatographic analysis of the *C. comosum* extract. (A) Total phenolic content (TPC) and total flavonoid content (TFC) of the *C. comosum* crude extract, expressed as mg gallic acid equivalent (GAE)/g extract and mg quercetin equivalent (QE)/g extract, respectively. Data are presented as mean ± standard deviation (*n* = 3); (B) HPLC chromatograms of the *C. comosum* aqueous extract. **1:** Gallic acid, **2:** Chlorogenic acid, **3:** Vanillic acid, **4:** Caffeic acid, **5:** Vanillin, **6:** P‐Coumaric acid, **7:** Rutin, **8:** Quercetin.

The extraction process yielded 1.069 g of dry extract from 10 g of *C. comosum* aerial parts, corresponding to an average yield of 10.69% ± 0.27%. This high yield demonstrates the efficiency of the aqueous maceration technique in extracting water‐soluble bioactive compounds under mild conditions, preserving their therapeutic potential. Qualitative analysis identified several bioactive compounds in the aqueous extract, including polyphenols, flavonoids, and saponins (Table 1). Quantitative analysis revealed a total phenolic content of 185.07 mg GAE/g extract and a total flavonoid content of 21.75 mg QE/g extract (Table 1), indicating notable antioxidant activity. HPLC analysis identified key antioxidant compounds, including quercetin (6499.76 μg/g), rutin (4618.41 μg/g), chlorogenic acid (677.15 μg/g), and gallic acid (529.05 μg/g) (Table 2 and Figure 2). These compounds are essential in combating oxidative stress and inflammation and may have potential therapeutic uses in managing chronic diseases like cardiovascular diseases and diabetes (Alghamdi et al. 2023). The abundant presence of quercetin and rutin highlights the extract's significant medicinal potential (Alehaideb et al. 2020). This phytochemical profile suggests that *C. comosum* could serve as an effective natural remedy to mitigate the oxidative stress and inflammation associated with PGB and validates its traditional use.

**TABLE 1:** Phytochemical screening and analysis of the *C. comosum* aqueous extract.

Qualitative analysis	
Phytochemical compounds	*C. comosum* (aqueous extract)
Polyphenols	(+)
Alkaloids (Mayer)	(−)
Alkaloids (Wagner)	(+)
Tannins (Catechin)	(+)
Tannins (Gallic)	(+)
Flavonoids	(+)
Saponins	(+)
Steroids	(+)
Terpenoids	(+)
**Quantitative analysis**	
TPC (mg GAE/g extract)	185.07 ± 4.0
TFC (mg QE/g extract)	21.7 ± 2.8

Abbreviations: (−) Absence; (+) Presence; TFC, total flavonoid content; TPC, total phenolic content.

**TABLE 2:** Retention times and concentrations of phenolic compounds detected in the aqueous extract of *C. comosum* using HPLC.

Peak no.	Phenolic compound	Retention time (min)	Concentration (μg/g extract)	PubChem CID
1	Gallic acid	5.29	529.05	149
2	Chlorogenic acid	13.39	677.15	1781
3	Vanillic acid	15.53	159.18	1568
4	Caffeic acid	16.27	201.62	1793
5	Vanillin	21.46	78.91	1064
6	p‐Coumaric acid	23.81	12.95	5,281,853
7	Rutin	28.37	4618.41	5,280,863
8	Quercetin	45.04	6499.76	5,280,343

*Note:* *y*, HPLC peak area; *x*, concentration (μg/mL).

### 3.2 *C. comosum* Sub‐Acute Toxicity

The sub‐acute toxicity evaluation of the *C. comosum* aqueous extract in Wistar albino rats indicated a positive safety profile. No significant adverse effects were observed at any of the administered doses (0, 100, 1000, and 2000 mg/kg) at 3, 24 h, 7 days, and 14 days post‐administration (*n* = 3 animals per dose) (Table 3). The rats maintained normal body weight, exhibited typical movement, had healthy eyes, and showed no signs of diarrhea or mortality. These results suggest that *C. comosum* extract is well‐tolerated, even at higher doses, consistent with previous research on plant extracts with high phenolic content, which typically exhibit low toxicity (Al‐Abrahim et al. 2013).

**TABLE 3:** Overview of the sub‐acute toxicity assessment of the *C. comosum* aqueous extract in Wistar albino rats.

Parameters	*C. comosum* dose			
	Control group (0 mg/kg)	Low dose group (100 mg/kg)	Medium dose group (1000 mg/kg)	High‐dose group (2000 mg/kg)
Body weight changes	Normal	Normal	Normal	Normal
Death	0	0	0	0
Movement	Normal	Normal	Normal	Normal
Diarrhea	Normal	Normal	Normal	Normal
Eyes	Normal	Normal	Normal	Normal

### 3.3 *C. comosum* Effect on Pregabalin‐Induced Toxicity

The impact of *C. comosum* extract on PGB‐induced toxicity was assessed in adult Wistar albino rats (*n* = 5 animals/group). The rats were assigned to one of four groups: untreated, treated with PGB (200 mg/kg body weight/day) via drinking water, treated with *C. comosum* extract (100 mg/kg body weight/day by gavage) for 35 days, or treated with PGB followed by *C. comosum* extract (100 mg/kg body weight/day by gavage) for the last 2 weeks.

#### 3.3.1 Body Weight Changes

**FIGURE 3:**
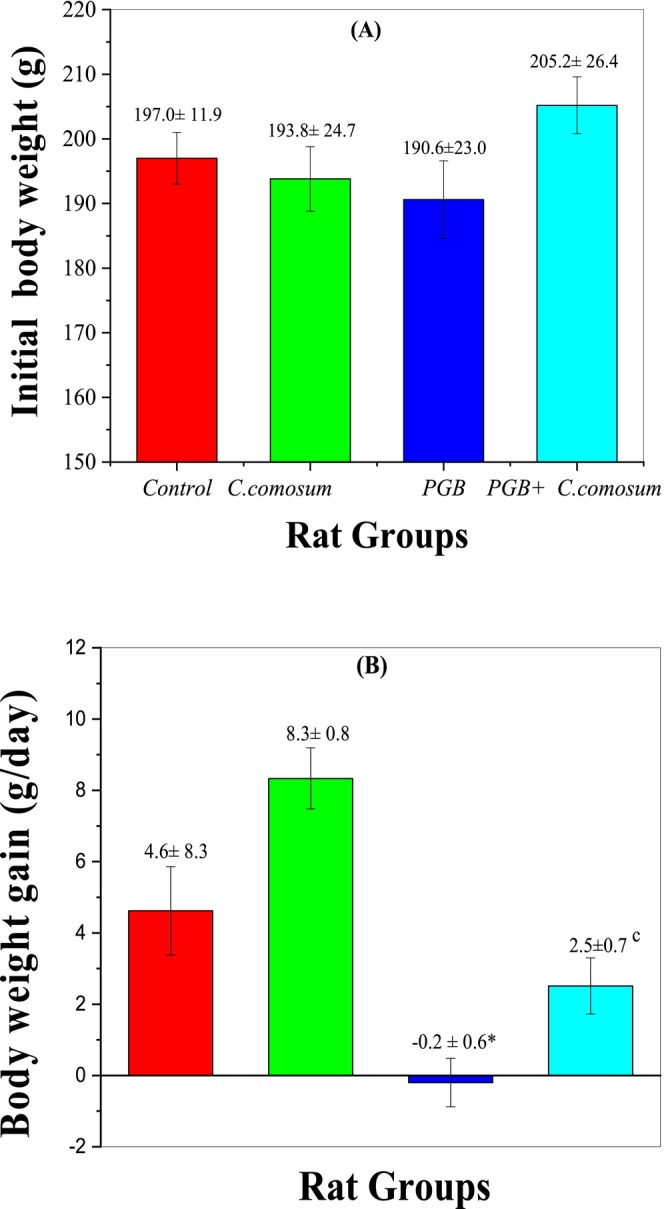
Change in body weight of the control group and the three treated rat groups. Data are presented as mean ± SEM (*n* = 5 per group). Statistical significance: *p < 0.05 versus Group I; ^*c*^ *p* < 0.001 versus Group III.

At the beginning of the study, body weight did not significantly differ among the four groups (*p* > 0.05, Figure 3A), indicating proper randomization. As shown in Figure 3B and detailed in Table S2, daily body weight monitoring revealed that Group 1 (untreated control) exhibited normal weight gain (4.6 ± 8.3 g/day). Similarly, rats in Group 2, which received *C. comosum* extract alone, showed a statistically comparable weight gain (8.3 ± 0.8 g/day, *p* > 0.05 vs. control). In contrast, Group 3 (PGB‐exposed rats) demonstrated a significant reduction in body weight (−0.2 ± 0.6 g/day), which was statistically significant compared to the control group (*p* < 0.05), likely due to PGB‐induced lipid degeneration, reduced food intake, and physical inactivity (Shokry et al. 2020). Remarkably, rats in Group 4 (PGB + *C. comosum* ) exhibited a partial recovery in body weight gain (2.5 ± 0.7 g/day), with a statistically significant improvement compared to the PGB group alone (*p* < 0.001), suggesting a potential protective effect of the plant extract.

The exclusive use of male rats, while aimed at reducing confounding hormonal influences, limits the extrapolation of the findings across sexes. Given known sex‐dependent differences in metabolism and endocrine responses, future investigations including female subjects are warranted to establish broader translational relevance (Perrino et al. 2021).

This suggests that the *C. comosum* extract may mitigate PGB‐induced weight loss and metabolic disturbances, potentially through activation of metabolic pathways (e.g., AMPK signaling) (Rzhepakovsky et al. 2022). These findings underscore the therapeutic potential of *C. comosum* in countering PGB's adverse effects, warranting more studies into the underlying mechanisms and its therapeutic applications.

#### 3.3.2 Relative Weight of Target Organs

At the end of the study, a comparative analysis of organ‐to‐body weight ratios revealed significant alterations across treatment groups. In the control group (Group 1), relative liver, kidney, and testis weights were within the normal physiological range (0.60 ± 0.01 g/100 g, 2.65 ± 0.05 g/100 g, and 2.03 ± 0.02 g/100 g, respectively). Similarly, rats administered *C. comosum* extract alone (Group 2) showed no significant changes in organ weights compared to the control (*p* > 0.05), confirming the extract's non‐toxic profile.

**FIGURE 4:**
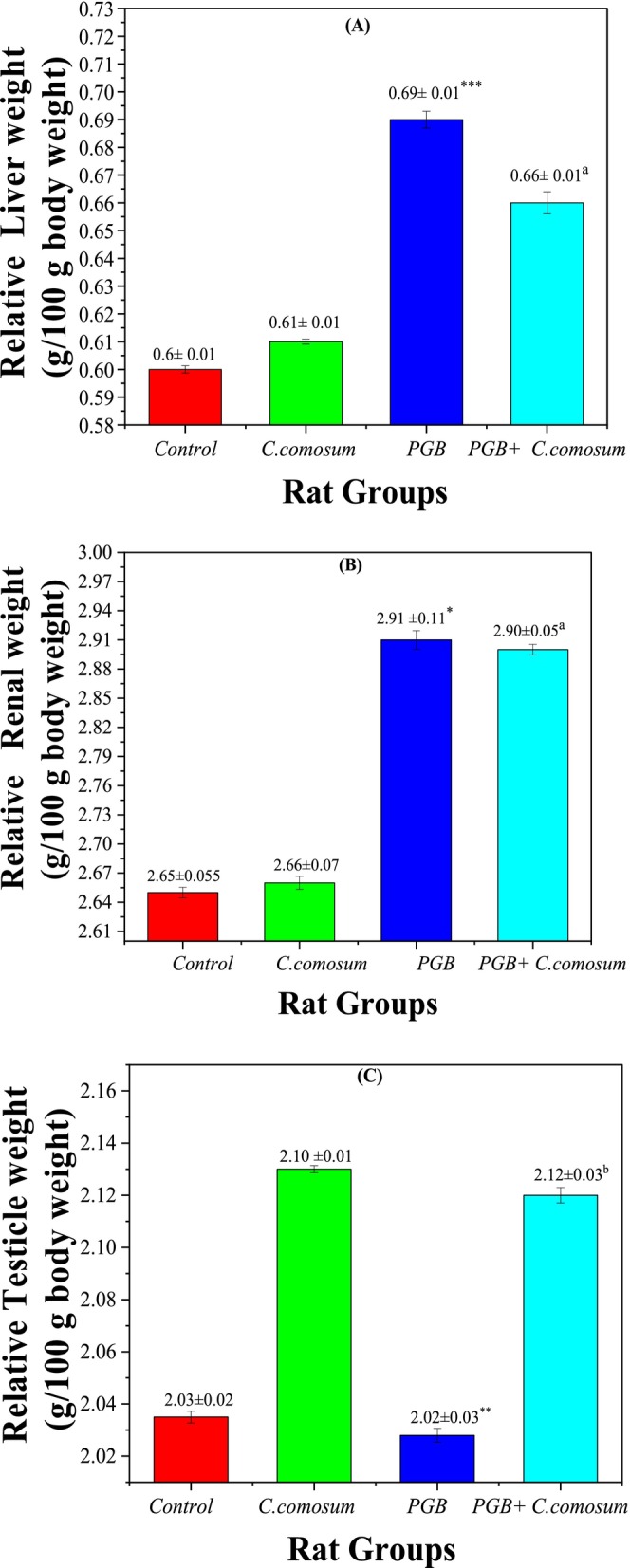
Relative organ weight of the control group and the three treated rat groups. (A) Relative liver weight (g/100 g body weight), (B) Relative kidney weight (g/100 g body weight), (C) Relative testis weight (g/100 g body weight). Data are presented as mean ± SEM (*n* = 5 per group). Statistical significance: *p < 0.05, **p < 0.01, ***p < 0.001 versus Group I; ^*a*^ *p* < 0.05, ^*b*^ *p* < 0.01 versus Group III.

In contrast, PGB‐treated rats (Group 3) exhibited a significant increase in liver weight (0.69 ± 0.01 g/100 g; *p* < 0.001 vs. control, Figure 4A) and kidney weight (2.91 ± 0.11 g/100 g; *p* < 0.05), suggesting hepatic hypertrophy and renal impairment. However, co‐administration of *C. comosum* following PGB exposure (Group 4) led to a partial reversal of these changes. Liver weight decreased significantly to 0.66 ± 0.01 g/100 g (*p* < 0.05 vs. Group 3), while kidney weight slightly declined (2.90 ± 0.05 g/100 g), indicating a mitigating effect of the extract against PGB‐induced organ damage. Additionally, testicular weight was significantly improved in Group 4 (2.12 ± 0.03 g/100 g) compared to Group 3 (1.98 ± 0.02 g/100 g), suggesting a potential protective role on reproductive organs. These findings underscore *C. comosum* 's therapeutic potential in counteracting PGB‐induced toxicity. The extract demonstrated significant promise in reducing liver and kidney damage, likely due to its potent antioxidant and anti‐inflammatory properties (Jakupov et al. 2023).

#### 3.3.3 Biochemical Parameters

**FIGURE 5:**
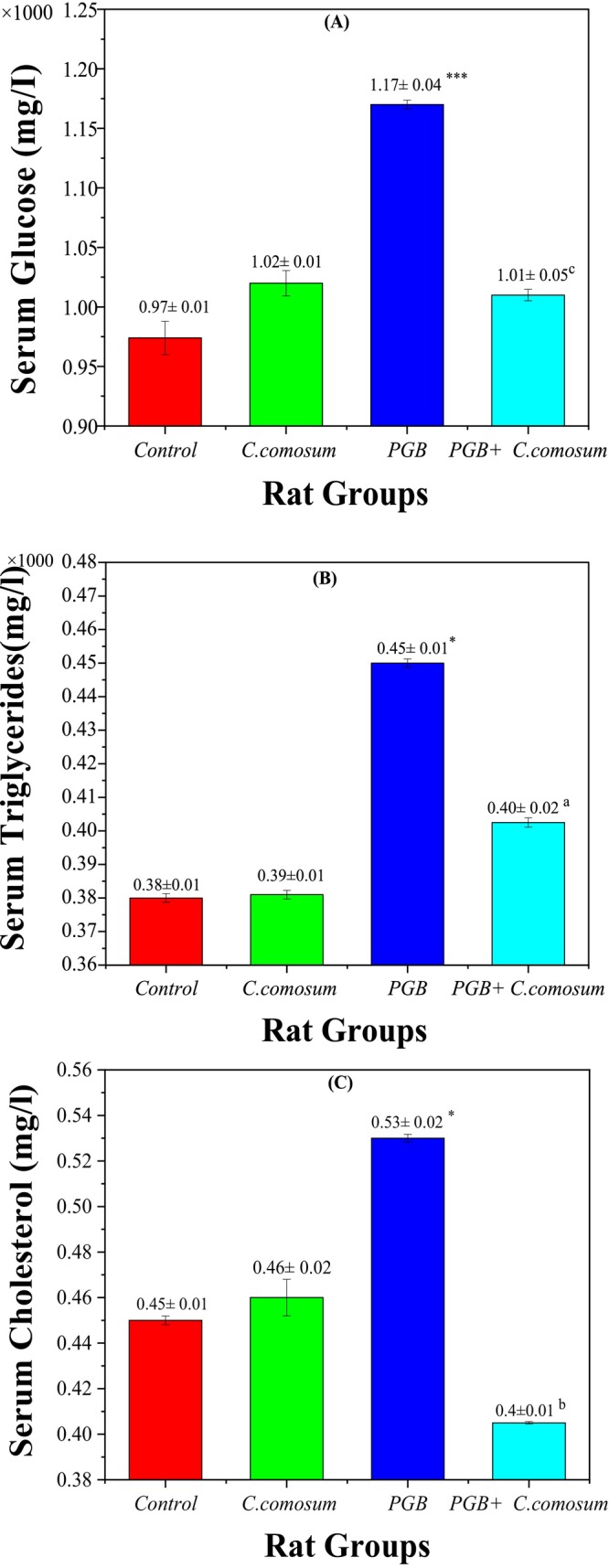
Serum biochemical parameters of the control group and the three treated rat groups. (A) Serum glucose (g/L), (B) Serum triglycerides (g/L), (C) Serum cholesterol (mg/L). Data are presented as mean ± SEM (*n* = 5 per group). Statistical significance: *p < 0.05, ***p < 0.001 versus Group I; ^a^ *p* < 0.05, ^b^ *p* < 0.01, ^c^ *p* < 0.001 versus Group III.

The biochemical analysis of serum triglycerides, cholesterol, and glucose levels showed significant alterations among the experimental groups (Figure 5 and Table S4). In the control group (Group 1), normal physiological levels were recorded for triglycerides (0.40 ± 0.01 × 1000 mg/L), cholesterol (0.47 ± 0.01 mg/L), and glucose (0.97 ± 0.01 × 1000 mg/L). Similarly, Group 2 (treated with *C. comosum* alone) exhibited no statistically significant changes in these parameters compared to controls (*p* > 0.05), confirming the metabolic safety of the extract.

However, PGB exposure (Group 3) significantly elevated all three parameters compared to the control group: triglycerides (0.42 ± 0.01 × 1000 mg/L, *p* < 0.05), cholesterol (0.50 ± 0.02 mg/L, *p* < 0.05), and glucose (1.12 ± 0.04 × 1000 mg/L, *p* < 0.001), suggesting dyslipidemia and hyperglycemia potentially driven by hepatic stress and insulin resistance. Remarkably, co‐administration of *C. comosum* following PGB exposure (Group 4) significantly improved these metabolic indicators: triglycerides (0.40 ± 0.02 × 1000 mg/L, *p* < 0.05 vs. Group 3), cholesterol (0.41 ± 0.01 mg/L, *p* < 0.01 vs. Group 3), and glucose (1.01 ± 0.05 × 1000 mg/L, *p* < 0.001 vs. Group 3). These results strongly support the ameliorative and regulatory role of *C. comosum* in reversing PGB‐induced metabolic disturbances (Jakupov et al. 2023; Tlili, Laib, Hammoudi, et al. 2024). These values were closer to control levels, demonstrating the extract's potential in mitigating PGB‐induced metabolic disturbances. The active compounds in *C. comosum* , such as tannic acid and naringenin, likely drive its therapeutic effects by enhancing glucose uptake, stimulating insulin secretion, protecting pancreatic *β* cells, and modulating lipid metabolism (Althurwi et al. 2023). These actions further support *C. comosum* as a promising agent for managing PGB‐induced hyperglycemia and hyperlipidemia, likely due to its antioxidant and anti‐inflammatory properties.

#### 3.3.4 Renal Function Biomarkers

**FIGURE 6:**
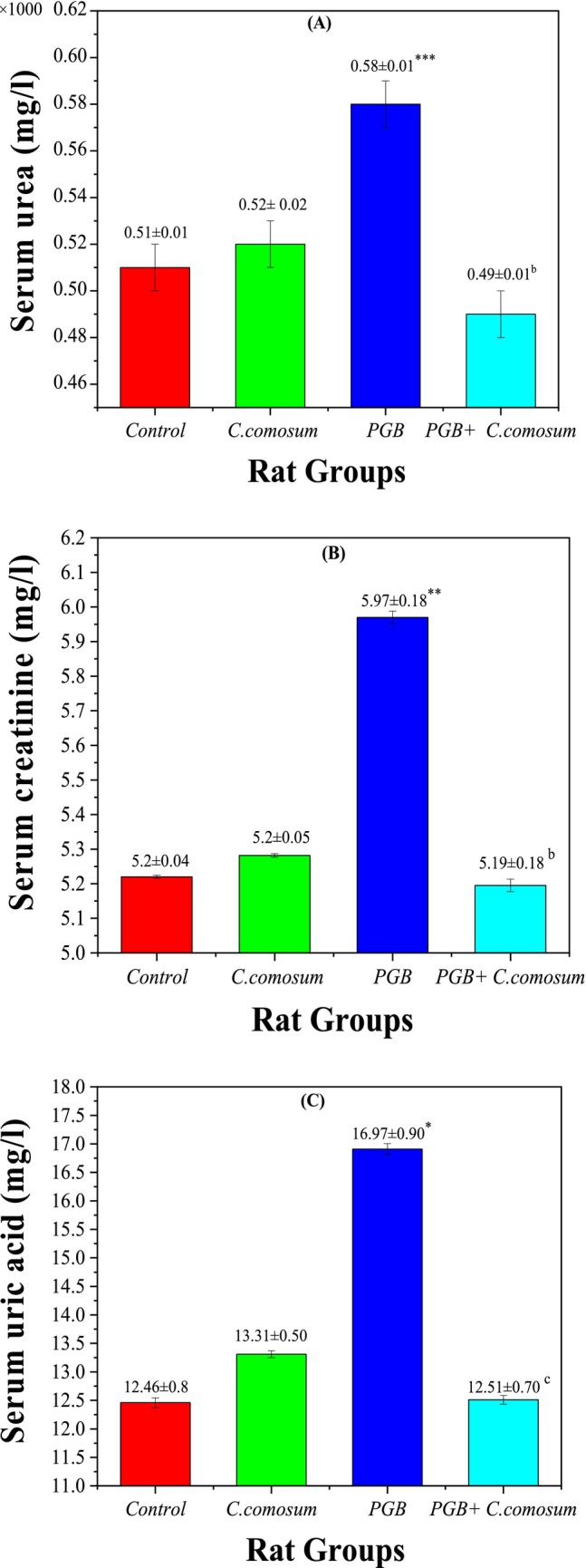
Kidney function biomarker concentrations in the control group and the three treated rat groups. (A) Serum urea (g/L), (B) Serum creatinine (mg/L), (C) Serum uric acid (mg/L). Data are presented as mean ± SEM (*n* = 5 per group). Statistical significance: *p < 0.05, **p < 0.01, ***p < 0.001 versus Group I; ^*b*^ *p* < 0.01, ^*c*^ *p* < 0.001 versus Group III.

Assessment of renal function biomarkers revealed clear evidence of PGB‐induced nephrotoxicity and the renoprotective effect of *C. comosum* treatment. In the control group (Group 1), serum urea (0.51 ± 0.01 × 1000 mg/L), creatinine (5.2 ± 0.04 mg/L), and uric acid (12.46 ± 0.80 mg/L) were within normal physiological ranges (Figure 6). Similarly, in Group 2 (rats treated with *C. comosum* alone), the biomarker levels of urea (0.48 ± 0.01 × 1000 mg/L), creatinine (5.28 ± 0.05 mg/L), and uric acid (11.92 ± 0.50 mg/L) did not significantly differ from those of the control group (*p* > 0.05), confirming the extract's biocompatibility and absence of nephrotoxic effects.

In contrast, Group 3 (PGB‐treated) showed a significant increase in all renal biomarkers compared to the control: serum urea (0.55 ± 0.01 × 1000 mg/L; *p* < 0.001), creatinine (5.43 ± 0.18 mg/L; *p* < 0.01), and uric acid (14.68 ± 0.90 mg/L; *p* < 0.05), indicating PGB‐induced nephrotoxicity.

Remarkably, co‐treatment with *C. comosum* after PGB exposure (Group 4) significantly restored renal biomarkers. Serum urea (0.51 ± 0.01 × 1000 mg/L; *p* < 0.01) and creatinine (5.19 ± 0.18 mg/L; *p* < 0.01) levels were significantly reduced compared to Group 3 (*p* < 0.01), and uric acid (10.28 ± 0.70 mg/L; *p* < 0.001) was also markedly decreased (*p* < 0.001). Moreover, no significant difference was observed between Group 4 and the control group for urea and creatinine levels (*p* > 0.05), indicating a complete functional recovery. These results support the strong nephroprotective potential of *C. comosum* , likely mediated by its antioxidant and anti‐inflammatory phytochemicals. This therapeutic effect is likely attributed to its antioxidant, anti‐inflammatory, and anti‐apoptotic properties, driven by bioactive compounds such as gallic acid and quercetin, which help improve renal function and reduce nephrotoxicity associated with PGB (Mbaveng et al. 2018).

#### 3.3.5 Liver Function Biomarkers

**FIGURE 7:**
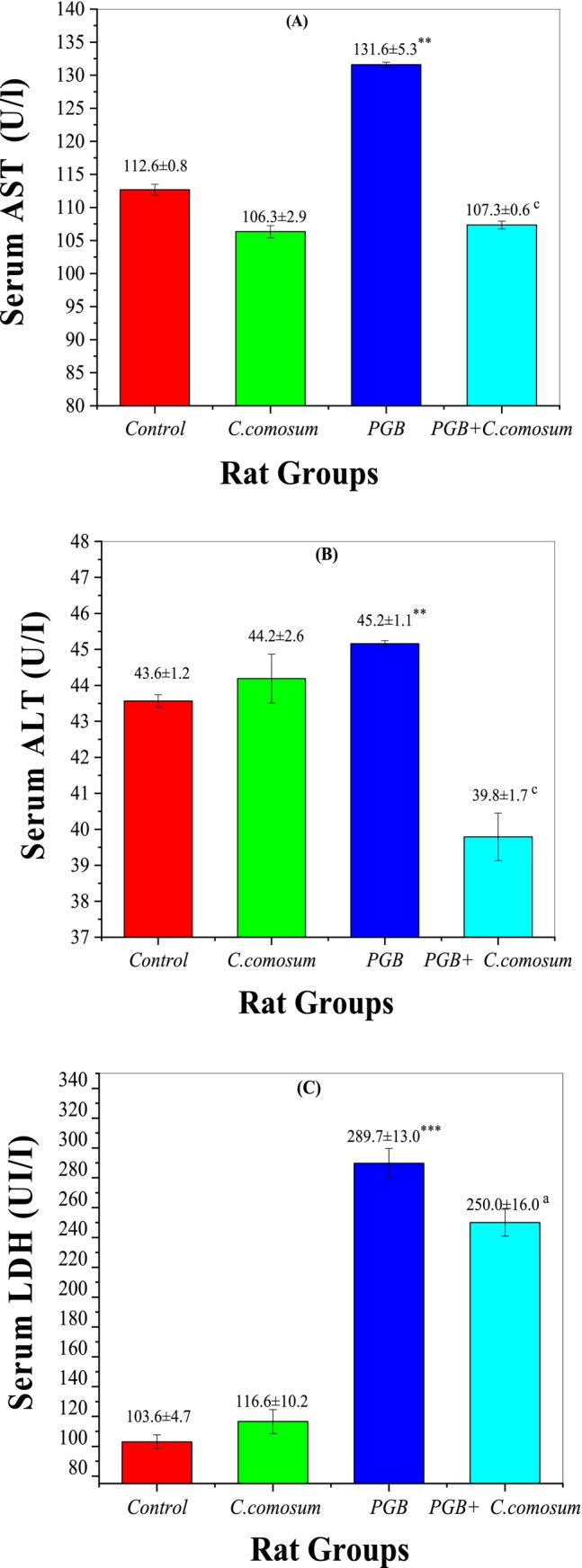
Liver function biomarkers in the control group and the three treated rat groups. (A) Serum AST (U/L), (B) Serum ALT (U/L), (C) Serum LDH (U/L). Data are presented as mean ± SEM (*n* = 5 per group). Statistical significance: **p < 0.01, ***p < 0.001 versus Group I; ^*a*^ *p* < 0.05, ^*c*^ *p* < 0.001 versus Group III.

Assessment of liver function biomarkers revealed significant evidence of PGB‐induced hepatotoxicity and the protective effects of *C. comosum* treatment (Figure 7). In the control group (Group 1), serum AST (112.6 ± 0.8 U/L), ALT (40.6 ± 44.2 U/L), and LDH (83.6 ± 4.7 U/L) were within normal physiological ranges. Similarly, in Group 2 (rats treated with *C. comosum* alone), the biomarker levels in particular AST (106.3 ± 4.9 U/L), ALT (44.2 ± 2.6 U/L), and LDH (116.6 ± 10.2 U/L) did not significantly differ from those of the control group (*p* > 0.05), confirming the extract's biocompatibility and absence of hepatotoxic effects.

In contrast, Group 3 (PGB‐treated) exhibited a significant increase in all liver biomarkers compared to the control group: serum AST (131.6 ± 5.4 U/L; *p* < 0.001), ALT (45.2 ± 1.1 U/L; *p* < 0.01), and LDH (289.7 ± 13.0 U/L; *p* < 0.001), indicating severe liver damage caused by PGB treatment, likely due to oxidative stress and inflammation (Mahdi et al. 2024).

Remarkably, co‐treatment with *C. comosum* after PGB exposure (Group 4) resulted in significant restoration of liver biomarkers. Serum AST (107.3 ± 0.6 U/L; *p* < 0.001), ALT (33.8 ± 1.7 U/L; *p* < 0.001), and LDH (250.0 ± 16.0 U/L; *p* < 0.05) levels were significantly reduced compared to Group 3, indicating partial recovery of liver function. These findings suggest that *C. comosum* helps alleviate PGB‐induced liver damage, possibly through its antioxidant and anti‐inflammatory effects (Alehaideb et al. 2020). This highlights *C. comosum* 's potential as a therapeutic agent against PGB‐induced hepatotoxicity.

#### 3.3.6 Hematological Parameters

**FIGURE 8:**
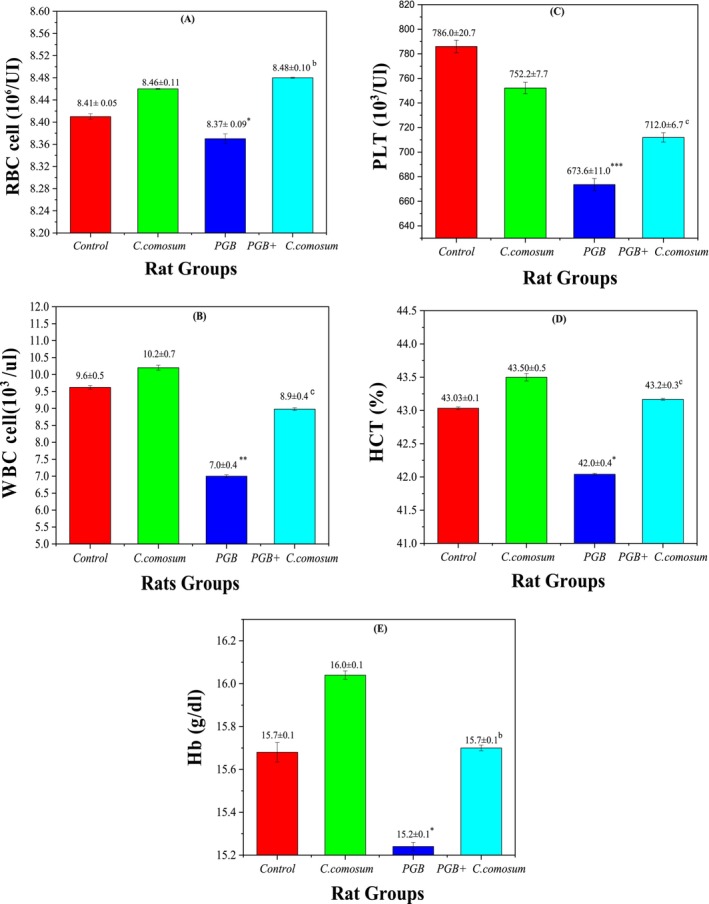
Hematological parameters of the control and treated rat groups. (A) Red Blood Cell (RBC) count (10^6^/μL), (B) White Blood Cell (WBC) count (10^3^/μL), (C) Platelet (PLT) count (10^3^/μL), (D) Hematocrit (HCT) percentage, and (E) Hemoglobin (Hb) concentration (g/dL). Data are presented as mean ± SEM (*n* = 5 per group). Statistical significance: *p < 0.05, **p < 0.01, ***p < 0.001 versus Group I, and ^a^ *p* < 0.05, ^b^ *p* < 0.01, ^c^ *p* < 0.001 versus Group III.

Evaluation of hematological parameters provided further insight into the protective effects of *C. comosum* against PGB‐induced hematotoxicity. In the control group (Group 1), all hematological values remained within normal physiological limits: RBC count (8.41 ± 0.05 × 10^6^/μL), WBC count (9.60 ± 0.50 × 10^3^/μL), HCT (43.0% ± 0.2%), hemoglobin concentration (15.7 ± 0.1 g/dL), and platelet count (786.0 ± 20.7 × 10^3^/μL), establishing baseline reference values (Figure 8).

Similarly, Group 2 (rats administered *C. comosum* alone) exhibited hematological values—RBC (8.46 ± 0.11 × 10^6^/μL), WBC (10.20 ± 0.70 × 10^3^/μL), HCT (43.5% ± 0.6%), Hb (16.0 ± 0.1 g/dL), and platelets (752.2 ± 7.7 × 10^3^/μL) that were not significantly different from the control group (*p* > 0.05), confirming the extract's hematological safety and biocompatibility.

In contrast, Group 3 (PGB‐treated) showed significant declines in all major hematological parameters: RBC count (8.37 ± 0.09 × 10^6^/μL; *p* < 0.05), WBC count (7.00 ± 0.40 × 10^3^/μL; *p* < 0.01), HCT (42.0% ± 0.5%; *p* < 0.05), Hb (15.2 ± 0.1 g/dL; *p* < 0.05), and platelet count (673.6 ± 11.0 × 10^3^/μL; *p* < 0.001), indicating PGB‐induced anemia, leukopenia, and thrombocytopenia. These hematological disturbances are likely due to oxidative damage and inflammation triggered by PGB, consistent with previous reports (El‐Sayed et al. 2019).

Remarkably, co‐treatment with *C. comosum* following PGB exposure (Group 4) led to significant improvements in all evaluated parameters: RBC (8.48 ± 0.10 × 10^6^/μL; *p* < 0.01 vs. Group 3), WBC (8.90 ± 0.40 × 10^3^/μL; *p* < 0.001), HCT (43.2% ± 0.4%; *p* < 0.001), Hb (15.7 ± 0.1 g/dL; *p* < 0.01), and platelets (712.0 ± 6.7 × 10^3^/μL; *p* < 0.001). These results underscore the hematoprotective effects of *C. comosum* , potentially attributed to its rich phytochemical content—including quercetin, gallic acid, and chlorogenic acid—which are known for their antioxidant and anti‐inflammatory activities (Abdo et al. 2015).

These findings suggest that *C. comosum* is a candidate therapeutic agent for managing drug‐induced hematological disorders, particularly those caused by PGB, and warrants further research for potential clinical applications.

#### 3.3.7 Testis Function Biomarkers

**FIGURE 9:**
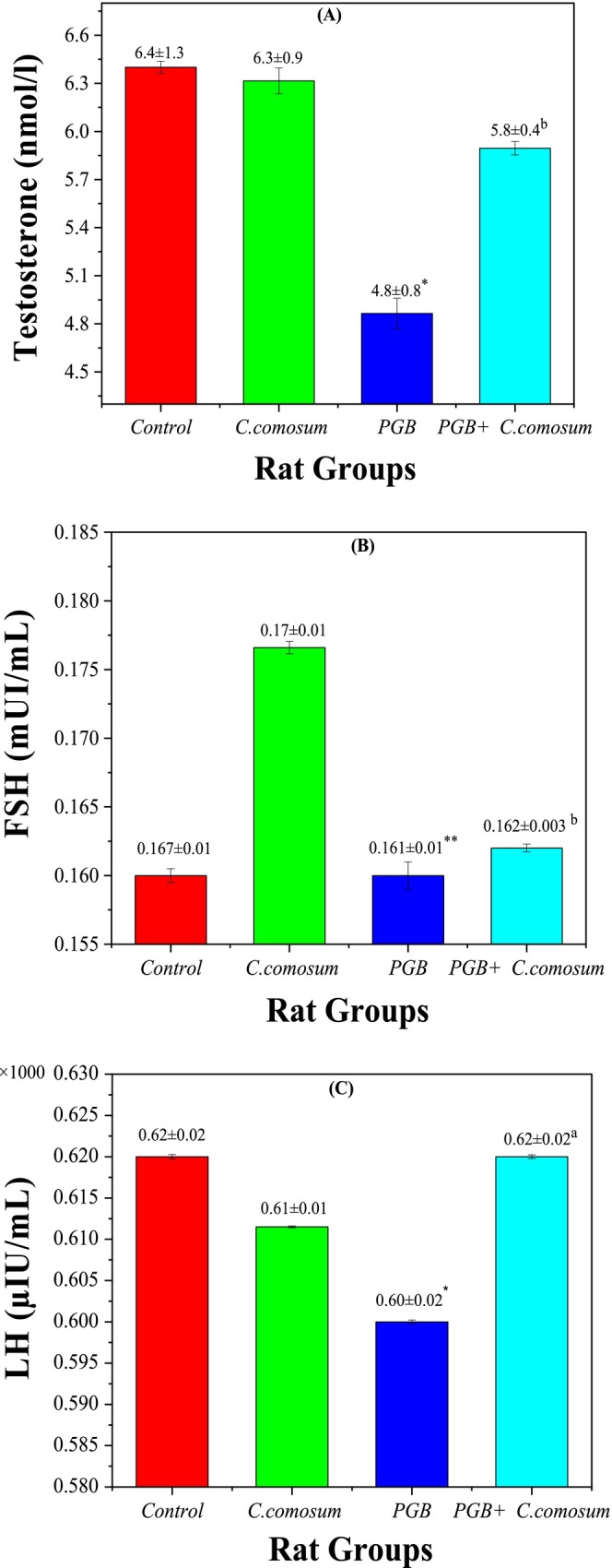
Testis function biomarkers in the control group and the three treated rat groups. (A) Testosterone (nmol/L), (B) FSH (mIU/mL), (C) LH (mIU/mL). Data are presented as mean ± SEM (*n* = 5 per group). Statistical significance: *p < 0.05, **p < 0.01 versus Group I; ^*a*^ *p* < 0.05, ^*b*^ *p* < 0.01 versus Group III.

Figure 9 and Table S8 illustrate the effects of PGB exposure and treatment with *C. comosum* extract on testis function (spermatogenesis) parameters. In Group 1 (Control), serum testosterone was 6.4 ± 1.3 nmol/L, FSH 0.167 ± 0.01 mIU/mL, and LH 0.62 ± 0.03 × 1000 μIU/mL, indicating normal testis function. Similarly, in Group 2 (rats treated with *C. comosum* alone), hormone levels including testosterone (6.3 ± 0.9 nmol/L), FSH (0.17 ± 0.01 mIU/mL), and LH (0.61 ± 0.01 × 1000 μIU/mL) showed no significant differences from the control group (*p* > 0.05). This confirms the extract's hormonal safety. It also suggests no adverse effects on reproductive function. In contrast, Group 3 (PGB‐treated) exhibited a marked decline in serum testosterone (4.8 ± 0.8 nmol/L; *p* < 0.05), FSH (0.161 ± 0.01 mIU/mL; *p* < 0.01), and LH (0.60 ± 0.02 × 1000μIU/mL; *p* < 0.05) compared to the control, suggesting that PGB disrupts the HPG axis and impairs reproductive hormone synthesis—likely through oxidative damage and inflammatory responses in the testes, consistent with previous reports (Morse 2016; Shokry et al. 2020). Notably, co‐treatment with *C. comosum* after PGB exposure (Group 4) resulted in a significant hormonal improvement: testosterone (5.8 ± 0.4 nmol/L; *p* < 0.01 vs. Group 3), FSH (0.162 ± 0.00289 mIU/mL; *p* < 0.01), and LH (0.62 ± 0.23 × 1000 μIU/mL; *p* < 0.05). This improvement suggests that *C. comosum* mitigates PGB‐induced hormonal imbalances through its potent anti‐inflammatory and restorative properties (Morse 2016).

#### 3.3.8 Histological Analysis of Liver, Kidney, and Testis Tissue Samples

**FIGURE 10:**
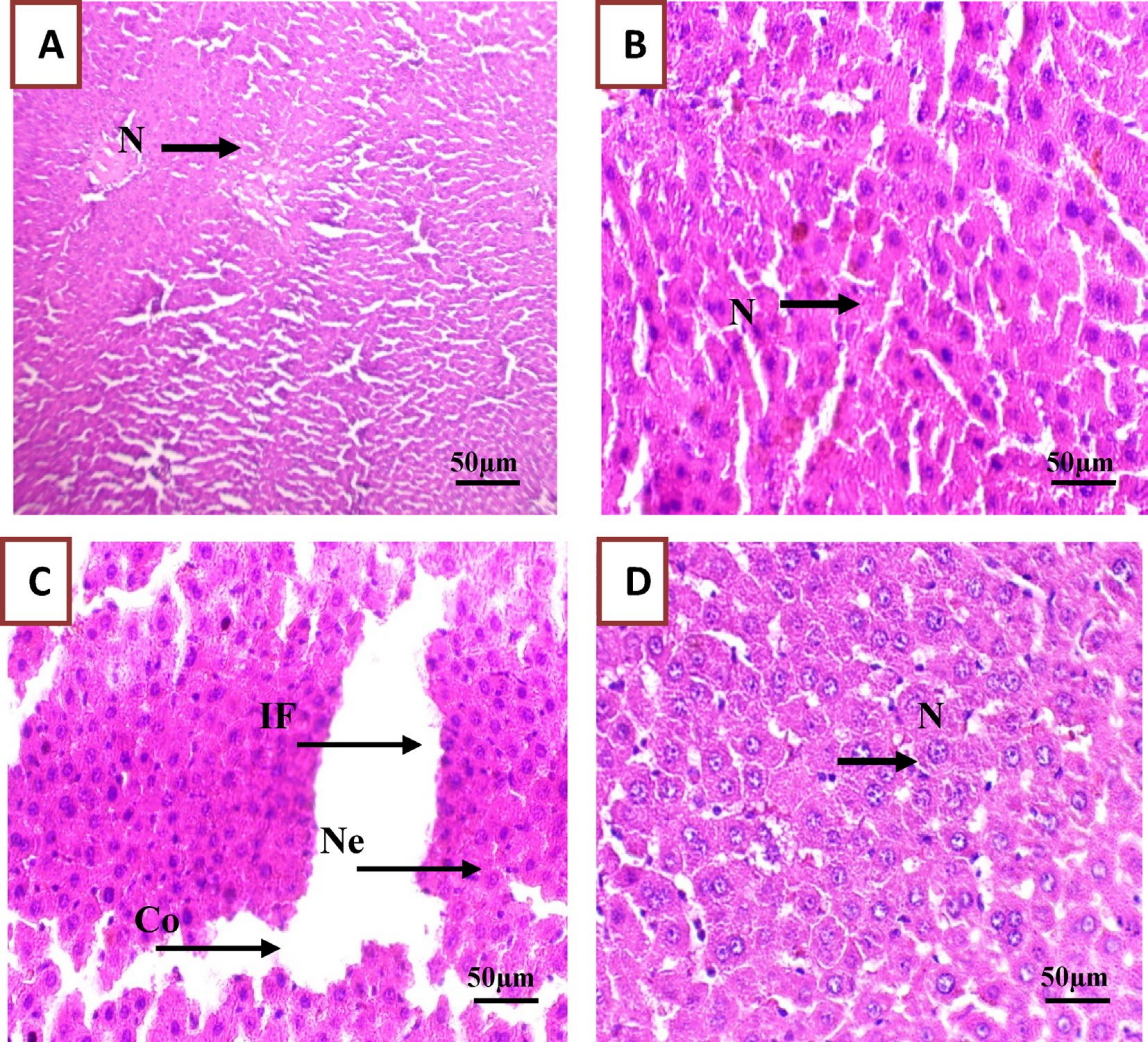
Figure: Representative micrographs of rat liver tissue sections from the experimental groups, highlighting the effect of PGB exposure and the protective role of *C. comosum* . (A) Group I (control); (B) Group II ( *C. comosum* ); (C) Group III (PGB); (D) Group IV ( *C. comosum* after PGB exposure). Co, Congestion; If, Inflammation; *N*, Normal cells; Ne, Necrosis; Scale bar = 50 μm, magnification × 40.

**FIGURE 11:**
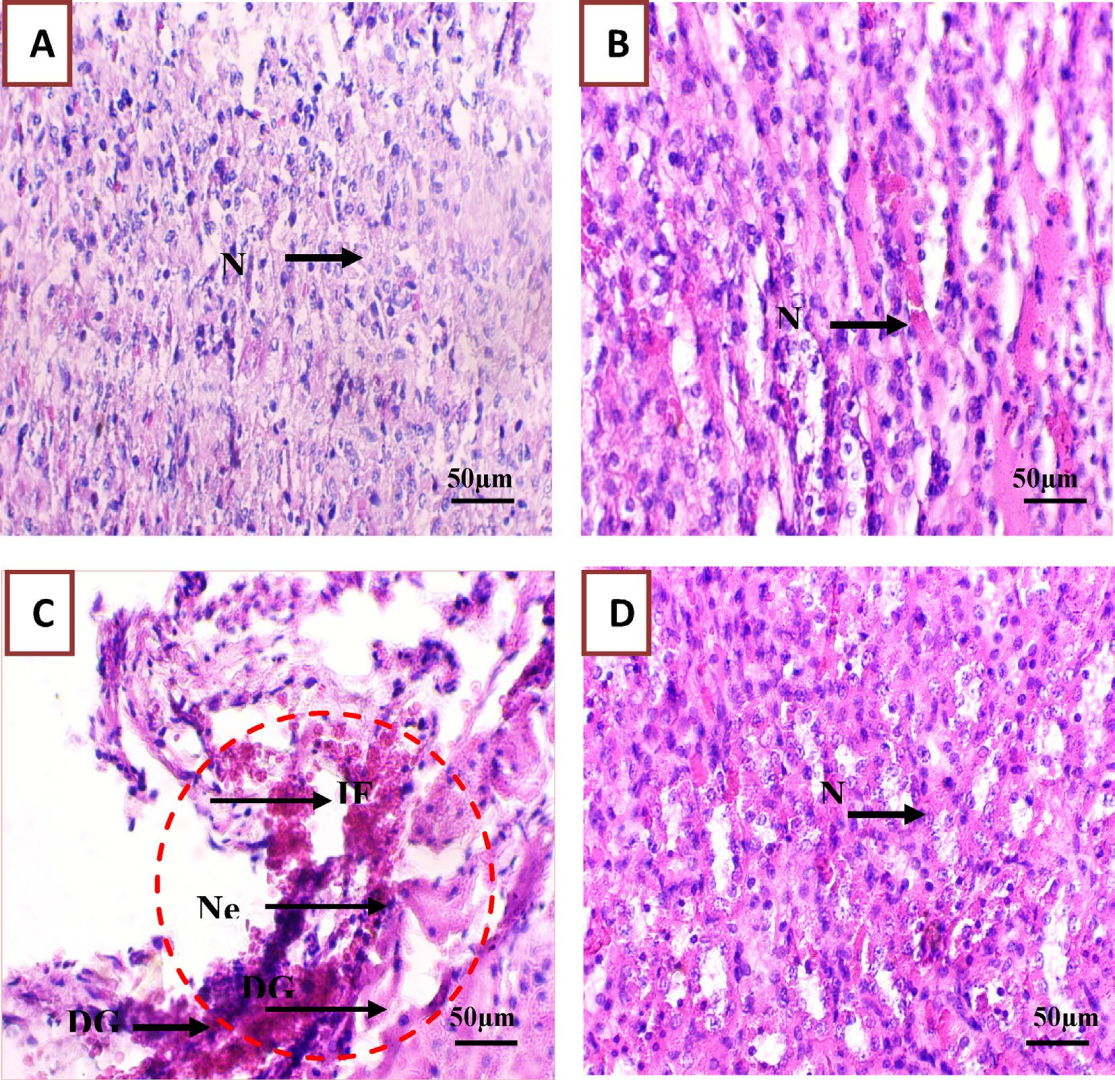
Representative micrographs of rat kidney tissue sections from various experimental groups, illustrating the impact of PGB exposure and the protective effect of *C. comosum* . (A) Group I (control); (B) Group II ( *C. comosum* ); (C) Group III (PGB); (D) Group IV ( *C. comosum* after PGB exposure). DG, Destroyed glomeruli; If, Inflammation; *N*, Normal cells; Ne, Necrosis; Scale bar = 50 μm, magnification × 40.

**FIGURE 12:**
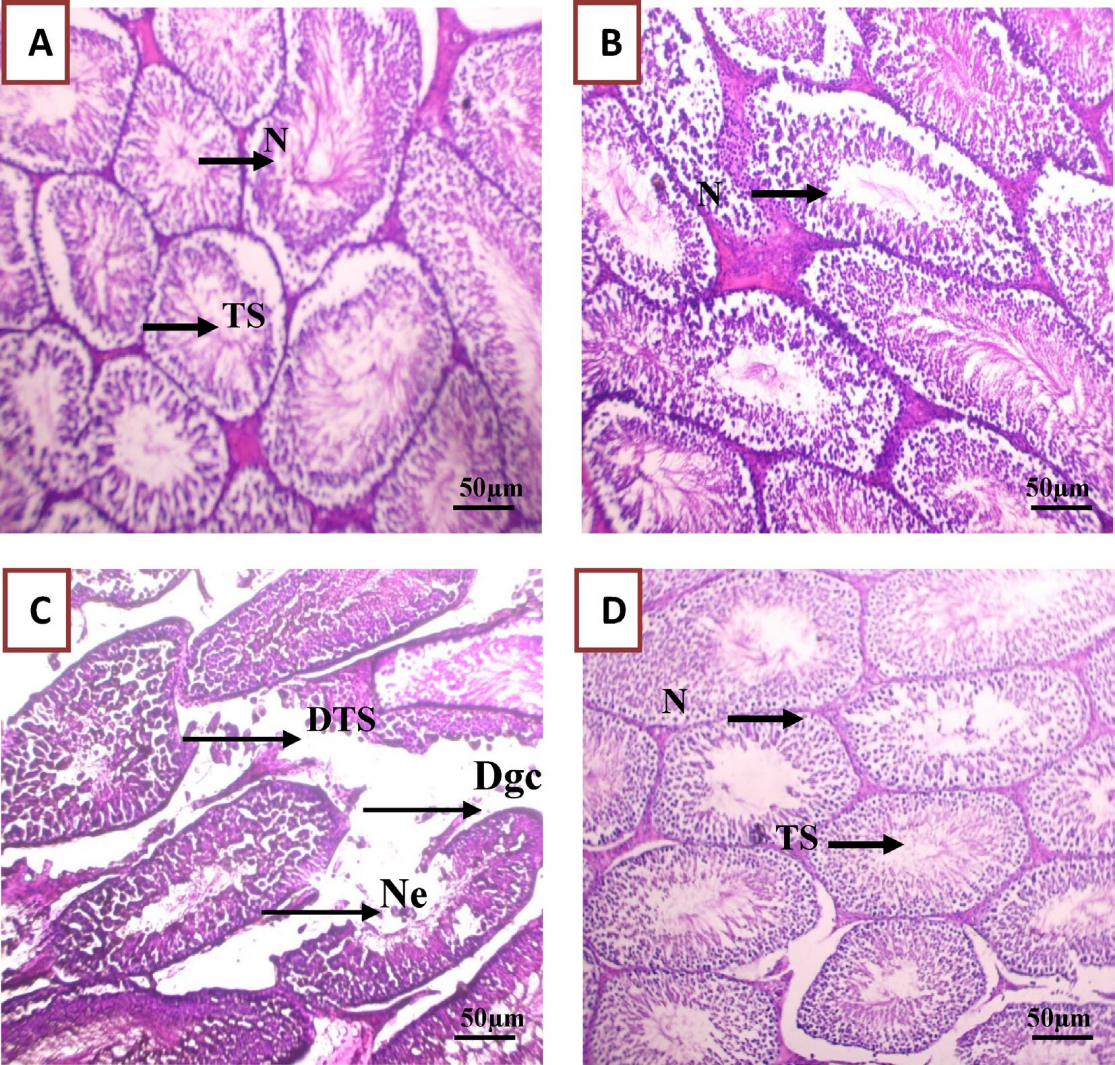
Representative images of rat testis tissue sections from the various experimental groups, illustrating the impact of PGB exposure and the protective effects of *C. comosum*. (A) Group I (control); (B) Group II ( *C. comosum* ); (C) Group III (PGB); (D) Group IV ( *C. comosum* after PGB exposure). Dg, Destroyed glomeruli; Dgc, Destruction of germ cells; DTS, Destroyed seminiferous tubules; *N*, Normal cells; Nc, Necrosis; Scale bar = 50 μm, magnification × 40; Ts, Seminiferous tubules.

The histological analysis at the study end (Figures 10, 11, 12) highlighted in liver samples from Group 3 (PGB), marked inflammatory infiltration (+++), hepatic necrosis (+), significant sinusoidal congestion (+++), and dilatation of hepatic veins (+++). Cytoplasmic vacuolation (+) indicated substantial cellular damage (Figure 10 and Table S9) (Ebrahem et al. 2022). Similarly, kidney samples from Group 3 displayed pronounced inflammatory cell (+++), glomerular destruction (+++), and extensive tubular dilation (+++), indicative of nephrotoxicity (Ebrahem et al. 2022; Morse 2016) (Figure 11 and Table S10). Testis damage in Group 3 included destruction of germ cells and seminiferous tubules, vascular congestion, and focal tissue necrosis (Figure 12 and Table S11), with severe disruption of the testis architecture (Salah et al. 2024).

Group 2 ( *C. comosum* extract) showed no significant histopathological change compared with the control group, indicating the extract's safety.

The reduced damage observed in Group 4 ( *C. comosum* after PGB exposure) compared to Group 3 can likely be attributed to the potent antioxidant effects of the extract, which contains bioactive compounds like gallic acid, quercetin, chlorogenic acid, and caffeic acid (Alghamdi et al. 2023). These phytochemicals are well‐established for their efficiency in neutralizing the free radicals and reactive oxygen species (ROS) generated by PGB. Previous studies have documented the protective action of *C. comosum* , which can be substantiated through histopathological analysis, revealing significantly lower levels of structural damage and inflammation markers in the organs of Group 4 compared to Group 3 (Abdel‐Sattar et al. 2019; Kiani et al. 2019). The extract's anti‐inflammatory and anti‐apoptotic effects, along with its ability to enhance blood circulation, play a critical role in minimizing tissue damage and promoting organ recovery. These findings align with results from studies by Aly et al. (2017) and Mohammad et al. (2023) (Aly et al. 2017; Mohammad et al. 2023), which highlight the diverse mechanisms by which *C. comosum* provides protection. Collectively, these experimental approaches offer robust evidence to support the hypothesis that *C. comosum* serves as an effective protector against PGB‐induced toxicity.

### 3.4 Molecular Docking Analysis and Binding Affinity

**FIGURE 13:**
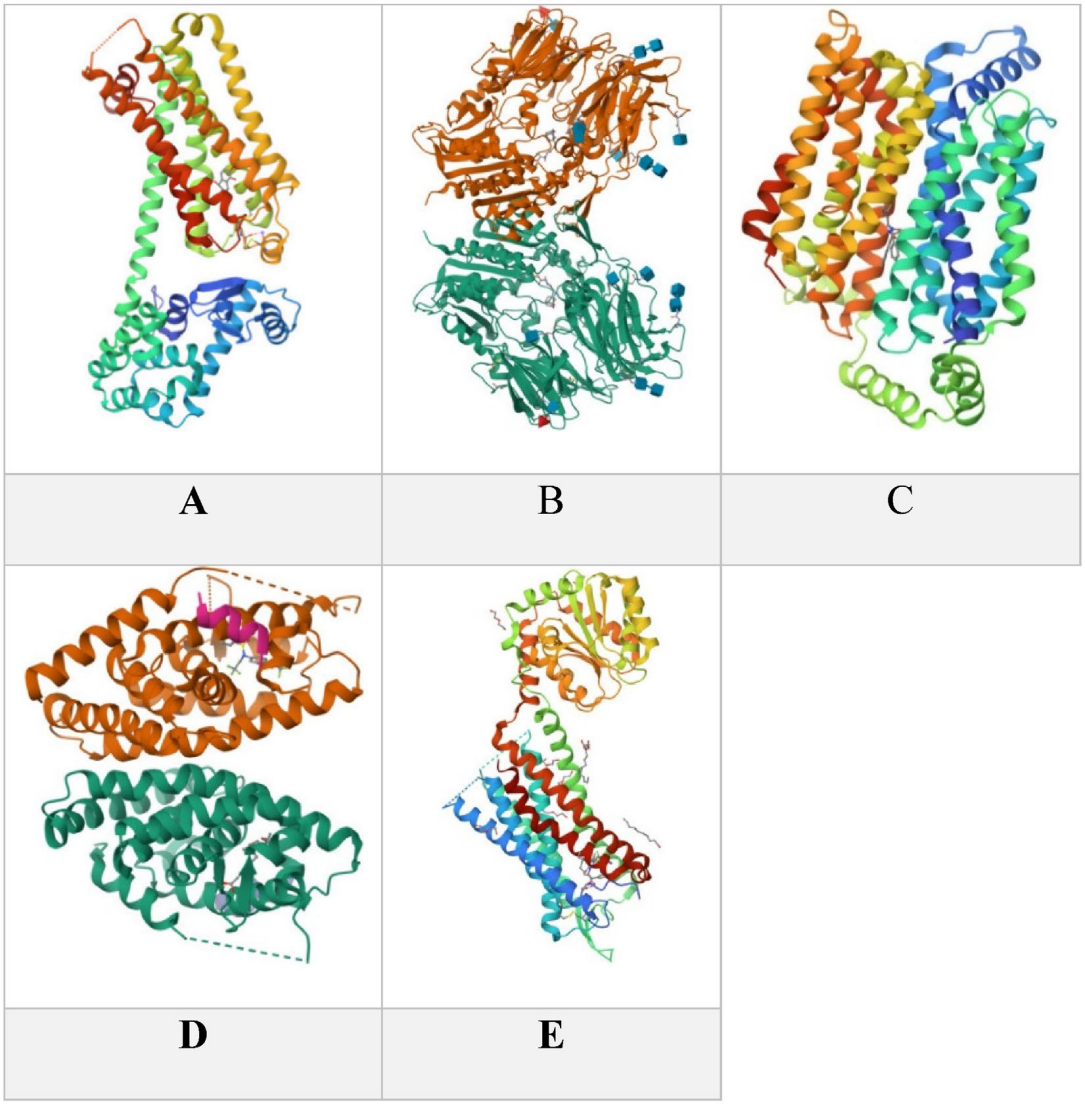
Crystallographic structure of common cell surface proteins: (A) Beta‐2 adrenergic receptor, (B) Dipeptidyl peptidase 4, (C) Glucose transporter 1, (D) Liver X receptor alpha, (E) Gonadotropin‐releasing hormone 1 receptor, with resolution of 3.1, 2.36, 2.9, 2.9, and 2.79 Å, respectively.

Then, molecular docking was used to evaluate and compare the binding affinity of PGB, a U.S. Food and Drug Administration‐approved anti‐epileptic drug (Morano et al. 2019), and of the *C. comosum* bioactive compounds, identified by HPLC, to the following targets: 2 AR (PDB ID: 7XKA), DPP‐IV (PDB ID: 3W2T), GLUT‐1 (PDB ID: 5EQG), LXR‐α (PDB ID: 1UHL), and GnRH1‐R (PDB ID: 7BR3). The crystallographic structures of these proteins are in Figure 13.

For the docking simulations, the Lamarckian genetic algorithm was used and the grid box coordinates for each target are listed in Table 3 (Morris et al. 1998). The biomolecule structures were converted into pdbqt format for docking after removing water and heteroatoms and adding polar hydrogens and Gasteiger charges. The docking results were validated using RMSD calculations, and values below 2 Å were considered indicative of good ligand–target interactions (Vianna and de Azevedo 2012).

PGB showed the strongest interaction with 2 AR (binding affinity of −6.0 kcal/mol) (Table 4), followed by DPP‐IV (−5.2 kcal/mol), GLUT‐1 (−5.1 kcal/mol), LXR‐α (−4.9 kcal/mol), and GnRH1‐R (−4.8 kcal/mol). However, PGB binding affinities were relatively lower compared with those of the *C. comosum* bioactive compounds (Table 5).

**TABLE 4:** Grid box centers and dimensions (in Å) of each target protein.

PDB (chain)	*x*‐center (dimensions)	*y*‐center (dimensions)	*z*‐center (dimensions)
7XKA (A)	−1.445 (40)	−11.800 (40)	−47.308 (40)
3W2T (A)	55.679 (50)	63.625 (40)	36.312 (40)
5EQG (A)	581.558 (40)	−25.192 (46)	277.963 (46)
1UHL (B)	76.188 (40)	−3.898 (40)	15.459 (40)
7BR3 (A)	−27.572 (40)	17.640 (50)	−9.562 (40)

**TABLE 5:** Binding affinity (kcal/mol) between target proteins and pregabalin or *C. comosum* bioactive compounds.

PDB ID	Quercetin	Rutin	Chlorogenic acid	Gallic acid	Caffeic acid	Pregabalin
7XKA	−8.2	5.3	−8.9	−7.1	−7.3	−6
3W2T	−8.2	−8.6	−7.9	−5.7	−6.1	−5.2
5EQG	−9.2	−10.1	−8.7	−6.1	−6.7	−5.1
1UHL	−8.1	−8.5	−8	−5.5	−5.6	−4.9
7BR3	−8.4	−10.4	−7.9	−6.0	−6.1	−4.8

Abbreviations: BE, binding energy in kcal/mol.; RMSD, root mean square distance.

For instance, quercetin showed strong interactions with all targets, with binding energies ranging from −8.1 (with LXR‐α) to −9.2 kcal/mol (with GLUT‐1) (Table 5, Figure S1). Rutin displayed the highest affinity for GnRH1‐R (−10.4 kcal/mol), and also a good affinity for GLUT‐1 (−10.1 kcal/mol). Caffeic acid, gallic acid, and chlorogenic acid exhibited strong binding affinity to the β2 AR with binding energies of −8.9 kcal/mol, −7.3 kcal/mol, and −7.1 kcal/mol, respectively (Table 5, Figures S1 and S2).

Molecular docking analysis revealed notable interactions between both ligands (Pregabalin and Rutin) and the selected protein targets (PDB IDs: 7XKA, 3W2T, 5EQG, 1UHL, and 7BR3), primarily through hydrogen bonding and hydrophobic interactions.

**FIGURE 14:**
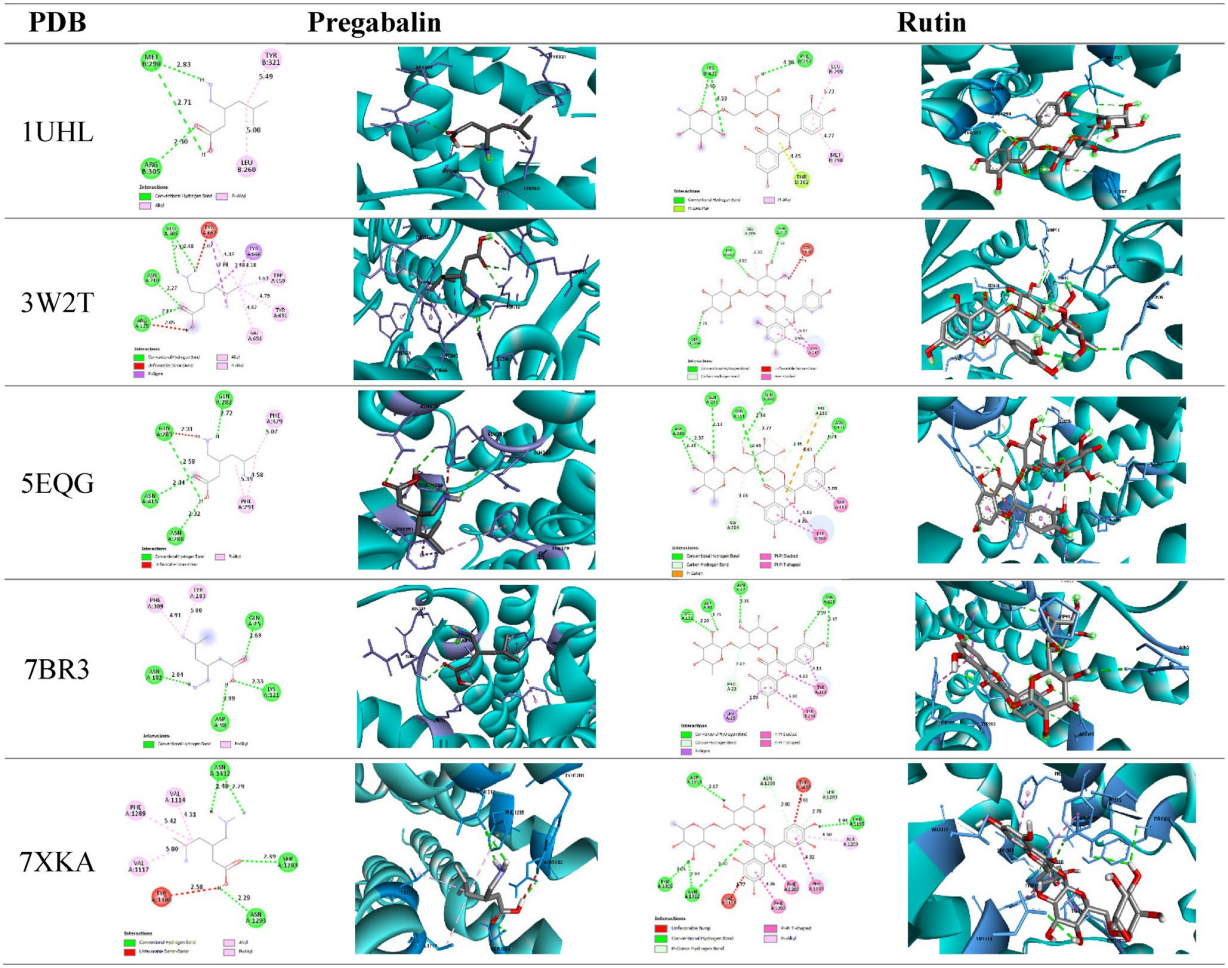
Interaction analysis of pregabalin and rutin with the selected targets (2D and 3D views). Protein targets are in cyan and interaction residues in light blue. Ligand atoms are: Gray: Carbon, Red: Oxygen, White: Hydrogen and Blue: Nitrogen.

Pregabalin exhibited stable binding across all targets. In the 7XKA complex, Pregabalin formed 4 hydrogen bonds with [Amino acid residues, Asn 1312 two bonds, Ser 1203, Asn 1293], effectively stabilizing the ligand within the binding pocket. For PDB ID 3W2T, it established 3 hydrogen bonds involving [residues, Glu 206 two bonds, Asn 710, Arg 125], indicating a favorable docking conformation. In the 5EQG structure, Pregabalin interacted via 4 hydrogen bonds with [residues, Gln 282, Gln 283, Asn 415, Asn 288], contributing to significant ligand affinity. Similarly, in PDB ID 1UHL, 3 hydrogen bonds were formed with [residues, Arg 305, and two bonds with Met 298], anchoring the ligand at the receptor interface. In the 7BR3 complex, Pregabalin maintained its binding through 4 hydrogen bonds with [residues, Gln 25, Asp 98, Asn 102, Lys 121], further suggesting high stability of the ligand–receptor interaction.

(Figure 14) shows the interactions between the targets and PGB and Rutin. PGB formed four conventional hydrogen bonds with β2‐adrenergic receptor (β2AR), specifically with Ser1203, Asn1293, and Asn1312, along with alkyl interactions involving Val1114, Phe1289, and Val1117. Despite these interactions, PGB binding energies were lower than those of the *C. comosum* compounds, particularly Rutin, as shown in (Table 5 and Figure 13).

Rutin, a polyphenolic flavonoid, demonstrated even stronger interaction profiles. In PDB ID 7XKA, Rutin formed 4 hydrogen bonds with [residues, Asp 113, Lys 1316, Asn 332, Arg 158], enhancing its binding affinity and orientation within the active site. For 3W2T, it exhibited 3 hydrogen bonds involving [residues, Asp 269, Lys 552, Ser 269], reflecting its high compatibility with the target protein. Within the 5EQG complex, Rutin engaged through 6 hydrogen bonds with [residues, Asn 411, Gln 161, Glu 380, Gln 283, two bonds with Asn 288], contributing to its robust docking stability. The interaction with PDB ID 1UHL involved three hydrogen bonds with [residues, Phe 257, and two bonds with His 421], while the 7BR3 structure revealed 5 hydrogen bonds between Rutin and [residues, Asn 27, Asp 98, Lys 121, and two bonds with Asn 305], further supporting its potential as a high‐affinity ligand.

The analysis indicated that elevated serum triglyceride and cholesterol levels in Group 3 suggest the drug may inhibit lipid metabolism signaling pathways. G protein‐coupled receptors (GPCRs), including beta‐adrenergic receptors (ARs), liver X receptors (LXRs), and farnesoid X receptors (FXRs), are key regulators of genes involved in lipid metabolism (Khan et al. 2015). DPP‐IV, a multifunctional enzyme present in various tissues, also influences this process by acting on incretin hormones like GLP‐1 and GIP, reducing insulin secretion (Andersen et al. 2018). Increased DPP‐IV expression in liver hepatocytes is a marker for liver disease and is linked to hepatic insulin resistance and steatosis (Shi et al. 2016). In type 2 diabetes, higher DPP‐IV activity in serum urea is a sign of diabetic nephropathy, a leading cause of end‐stage renal disease (ESRD) (Hasan and Hocher 2017). The bioactive compounds in *C. comosum* extract show potential as a therapeutic agent against pregabalin‐induced side effects due to their synergistic effects, offering a promising alternative treatment.

## 4 Conclusion

This study highlights the therapeutic potential of *C. comosum* aqueous extract in mitigating pregabalin (PGB)‐induced toxicity in male Wistar rats, with a particular emphasis on hepatic, renal, and reproductive health. PGB exposure caused significant biochemical disturbances, including elevated liver enzymes, impaired kidney markers, and hormonal imbalances, alongside substantial histopathological damage in the liver, kidneys, and testicular tissues. Treatment with *C. comosum* extract significantly restored liver and kidney functions, evidenced by reductions in liver enzymes (AST, ALT, and LDH) of 18.5%, 25.2%, and 13.7%, respectively, and a substantial improvement in kidney biomarkers (urea, creatinine, and uric acid), with reductions of 30.3%, 38.0%, and 15.2%. Furthermore, reproductive recovery was suggested by improvements in testosterone and LH levels, demonstrating the extract's role in restoring hormonal balance. Histological analyses supported these biochemical improvements, revealing reduced inflammation, necrosis, and congestion in treated tissues, further confirming the protective effects of *C. comosum* against organ damage induced by PGB. In silico molecular docking studies provided additional insights, showing that key phytochemicals from *C. comosum* , particularly quercetin, rutin, and caffeic acid, exhibited strong binding interactions with molecular targets related to oxidative stress, inflammation, and toxicity pathways, such as LXR‐α, GLUT‐1, and GnRH1‐R, with quercetin showing the strongest binding affinity. These findings collectively underline the ability of *C. comosum* to counteract the toxic effects of PGB on vital organs, providing a promising natural therapeutic alternative for mitigating drug‐induced hepatorenal and reproductive toxicity. The integration of experimental and computational approaches strengthens the validity of the results, suggesting that *C. comosum* could play a significant role in protecting against drug‐induced toxicity and promoting organ health. However, further studies in chronic models and clinical settings are necessary to fully confirm its potential for therapeutic use.

## Author Contributions

**Smail Mehda:** conceptualization (equal), investigation (equal), resources (equal), software (equal), validation (equal). **Ibtissam Laib:** conceptualization (equal), formal analysis (equal), investigation (equal), methodology (equal), resources (equal), software (equal), software (equal), supervision (equal), supervision (equal), validation (equal), validation (equal), visualization (equal), writing – original draft (equal), writing – original draft (equal), writing – review and editing (equal), writing – review and editing (equal). **Feriel Diab:** formal analysis (equal), investigation (equal), resources (equal), software (equal), supervision (equal). **Raounek Attia:** conceptualization (equal), data curation (equal), formal analysis (equal), funding acquisition (equal), software (equal), supervision (equal), validation (equal), visualization (equal). **Yousef Benaissa:** conceptualization (equal), data curation (equal), formal analysis (equal), resources (equal), software (equal), supervision (equal), validation (equal). **Attia Hanane:** conceptualization (equal), validation (equal), visualization (equal). **Khiari Rayhana:** conceptualization (equal), data curation (equal), supervision (equal). **Meriem Bellabidi:** conceptualization (equal), data curation (equal), resources (equal). **Huda Alsaeedi:** data curation (equal), formal analysis (equal), funding acquisition (equal), resources (equal). **David Croun:** conceptualization (equal), data curation (equal), formal analysis (equal), resources (equal), validation (equal). **Mikhael Bechelany:** conceptualization (equal), data curation (equal), formal analysis (equal), funding acquisition (equal), investigation (equal), resources (equal). **Ahmed Barhoum:** conceptualization (equal), data curation (equal), formal analysis (equal), funding acquisition (equal), investigation (equal), methodology (equal), resources (equal), software (equal), supervision (equal), validation (equal), visualization (equal), writing – original draft (equal), writing – review and editing (equal).

## Ethics Statement

All procedures followed the institutional and ethical standards approved by the El Oued University Ethics Committee (Approval No. 10/S.C./FL/NS/EU/2024).

## Consent

Informed consent was obtained from all participants included in the study, ensuring their voluntary participation. All authors have agreed to the publication of this manuscript and consented to its content and findings being shared.

## Conflicts of Interest

The authors declare no conflicts of interest.

## Supporting information


Appendix S1.

## Data Availability

The datasets generated and/or analyzed during this study are available as Supporting Information or can be obtained from the corresponding author upon reasonable request.
