# Supplementary material for: Protective Effects of Calligonum comosum as a Natural Remedy to Counteract Pregabalin‐Induced Toxicity: Insights From Chemical Profiling, In Vivo, and In Silico Analyses
Source: Food Sci Nutr. 2025 Jul 25;13(7):e70681. doi: 10.1002/fsn3.70681 (PMC12290306; doi:10.1002/fsn3.70681)
Supplement: Supplementary file 1 — Appendix S1. [file FSN3-13-e70681-s001.docx]

Protective Effects of *Calligonum comosum* as a Natural Remedy to Counteract Pregabalin-Induced Toxicity: Insights from Chemical Profiling, *In Vivo*, and *In Silico* Analyses

Smail Mehda^1,2^, Ibtissam Laib^2,3^, Feriel Diab^3^, Raounek Attia^3^, Yousef Benaissa^4^, Attia Hanane^3^, Khiari Rayhana^3,^ Meriem Bellabidi^5^, Huda Alsaeedi^6^, David Croun^7^, Mikhael Bechelany ^7,8^, Ahmed Barhoum^9,10*^

^1^ Faculty of Life and Natural Sciences, Department of Agronomy, University of El Oued, El Oued,39000, Algeria; [mehda-smail@univ-eloued.dz](mailto:mehda-smail@univ-eloued.dz)

^2^ Laboratory of Biodiversity and Biotechnology Applications in Agriculture, University of El Oued, 39000, Algeria

^3^ Department of Cellular and Molecular Biology, El Oued University, Algeria; [laib-ibtissam@univ-eloued.dz](mailto:laib-ibtissam@univ-eloued.dz4), [ferieldiab87@gmail.com](mailto:ferieldiab87@gmail.com), [Hananeatia@gmail.com](mailto:Hananeatia@gmail.com), [KhiariRayh@gmail.com](mailto:KhiariRayh@gmail.com)

^4^ VPRS Laboratory, Chemistry Department, Faculty of Mathematics and Matter Sciences. University of KASDI Merbah, Ouargla 30000, Algeria; [bay1995.h@gmail.com](mailto:bay1995.h@gmail.com)

^5^ Higher School of Saharan Agriculture, El Oued, Algeria; Meriam@gmail.com

^6^Department of Chemistry, College of Science, King Saud University, Riyadh, Saudi Arabia; [halsaeedi@ksu.edu.sa](mailto:halsaeedi@ksu.edu.sa)

^7^Institut Européen des Membranes, IEM, UMR-5635, Univ Montpellier, ENSCM, CNRS, Place Eugene Bataillon, 34095 Montpellier, France; [david.cornu@umontpellier.fr](mailto:david.cornu@umontpellier.fr), [mikhael.bechelany@umontpellier.fr](mailto:mikhael.bechelany@umontpellier.fr)

^8^ Functional Materials Group, Gulf University for Science and Technology (GUST), Mubarak Al-Abdullah 32093, Kuwait

^9^ NanoStruc Research Group, Chemistry Department, Faculty of Science, Helwan University, Cairo, 11795, Egypt

^10^ Chemical and BioPharmaceutical Sciences, Technological University Dublin, Grangegorman Campus, 7, Dublin, D07 ADY7, Ireland

* Corresponding Author: [ahmed.barhoum@science.helwan.edu.eg](mailto:ahmed.barhoum@science.helwan.edu.eg), [ahmed.barhoum@tudublin.ie](mailto:ahmed.barhoum@tudublin.ie)

**Supplementary information**

| PDB | Quercetin | | Gallic Acid | |
| --- | --- | --- | --- | --- |
| 7XK A | 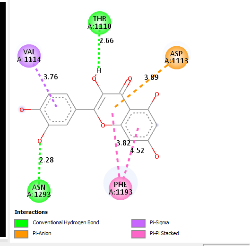 | 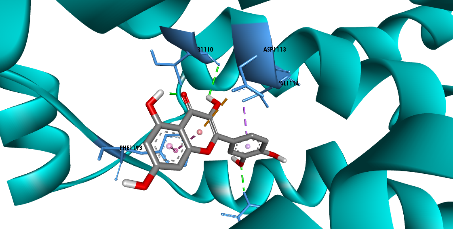 | 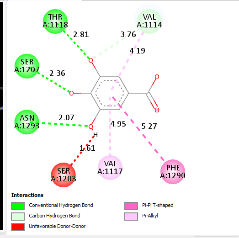 | 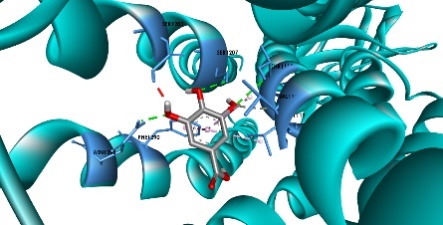 |
| 3W2T | 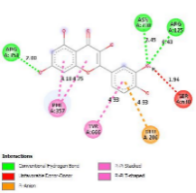 | 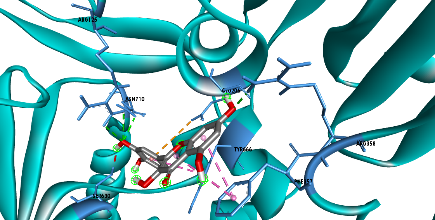 | 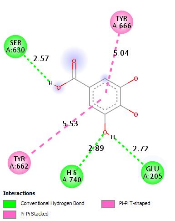 | 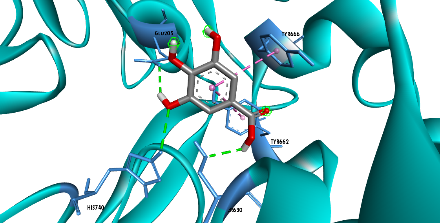 |
| 5EQG | 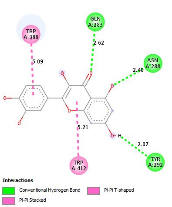 | 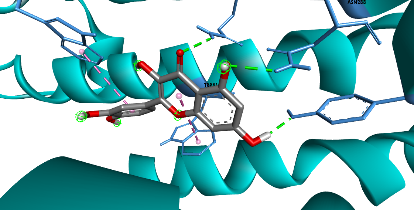 | 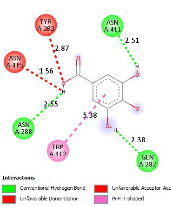 | 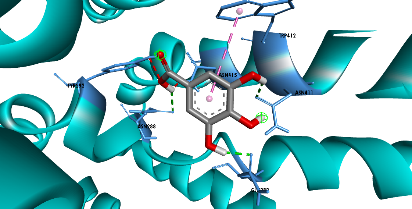 |
| 1UHL | 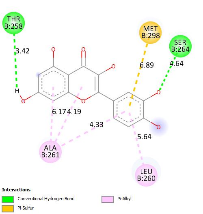 | 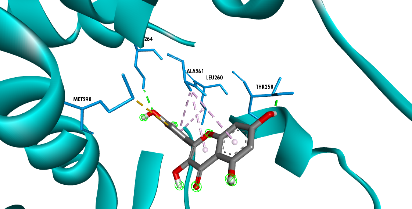 | 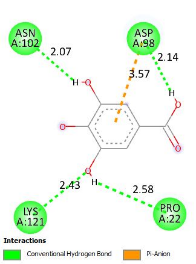 | 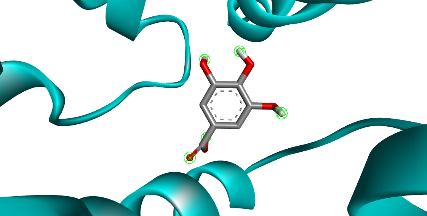 |
| 7BR3 | 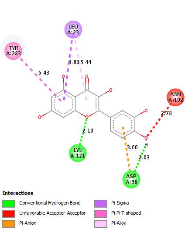 | 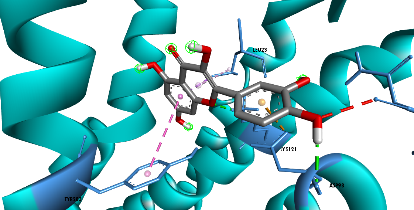 | 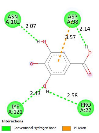 | 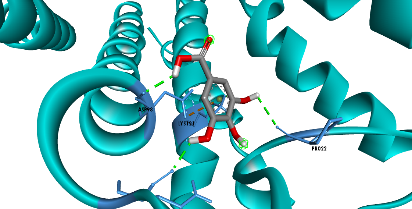 |

Figure S1. Interactions between quercetin and gallic acid with the indicated targeted proteins in 2D (left) and 3D (right). Targets are in cyan and interactions residues in light blue. 7XKA, β2 adrenergic receptor; 3W2T, dipeptidyl peptidase 4; 5EQG, glucose transporter 1; 1UHL, liver X receptor alpha; and 7BR3, gonadotropin-releasing hormone 1 receptor.

| PDB | Chlorogenic acid | | Caffeic acid | |
| --- | --- | --- | --- | --- |
| 7XKA | 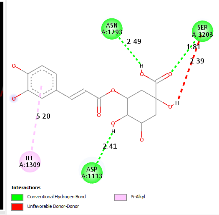 | 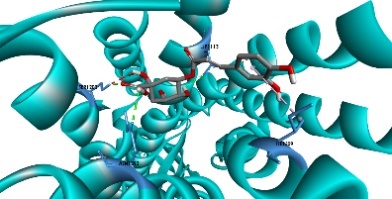 | 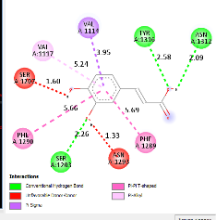 | 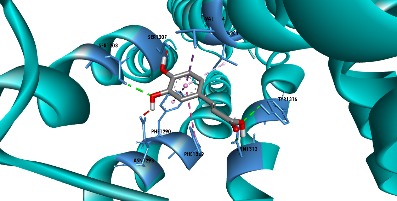 |
| 3W2T | 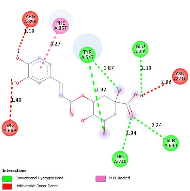 | 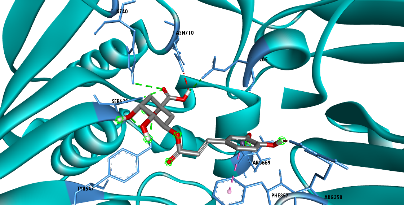 | 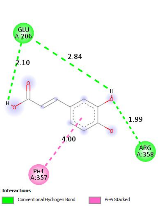 | 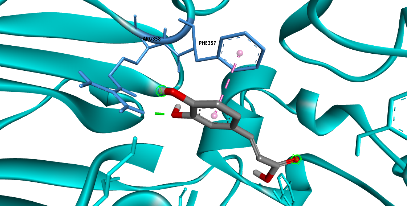 |
| 5EQG | 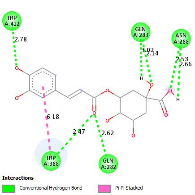 | 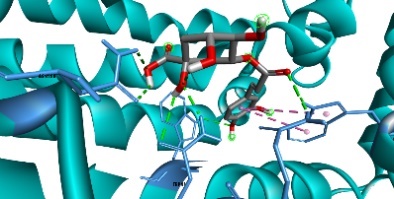 | 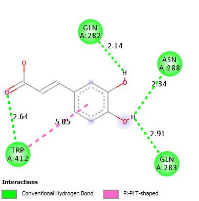 | 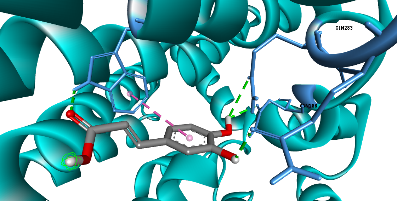 |
| 1UHL | 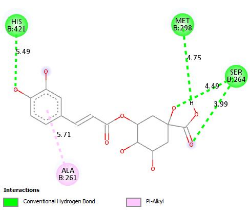 | 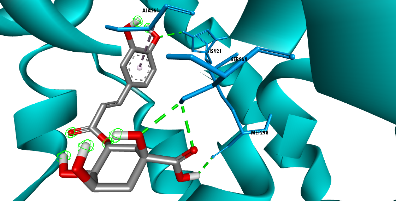 | 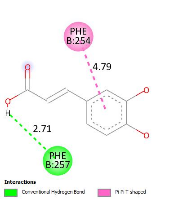 | 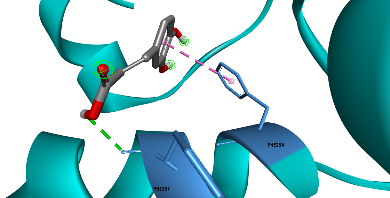 |
| 7BR3 | 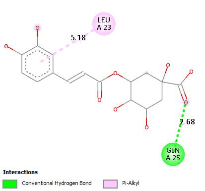 | 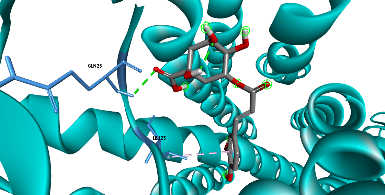 | 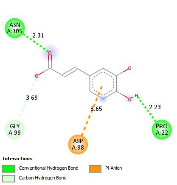 | 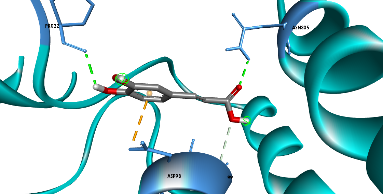 |

Figure S2. Interactions between chlorogenic acid and caffeic acid with the indicated targeted molecules in 2D (left) and 3D (right). Targets are in cyan and interactions residues in light blue. 7XKA, β2 adrenergic receptor; 3W2T, dipeptidyl peptidase 4; 5EQG, glucose transporter 1; 1UHL, liver X receptor alpha; and 7BR3, gonadotropin-releasing hormone 1 receptor.

Table S1. RMSD and binding energy (BE) values between ligands and their targeted molecules. Superpose of re-docked (orange) and original co-crystals (green). 7XKA, β2 adrenergic receptor; 3W2T, dipeptidyl peptidase 4; 5EQG, glucose transporter 1; 1UHL, liver X receptor alpha; and 7BR3, gonadotropin-releasing hormone 1 receptor.

| PDB ID | Co-crystal ligand | | RMSD | Co-crystal superpose |
| --- | --- | --- | --- | --- |
|  | Structure | BE |  |  |
| 7XKA | 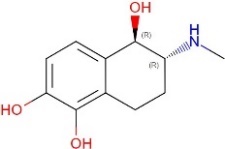 | -7.8 | 0.4616 | 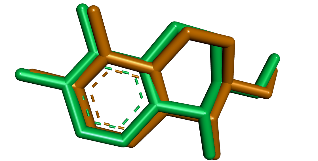 |
| 3W2T | 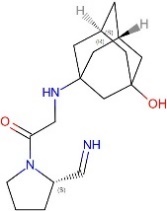 | -6.6 | 2.9684 | 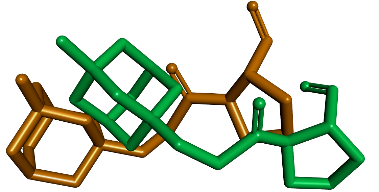 |
| 5EQG | 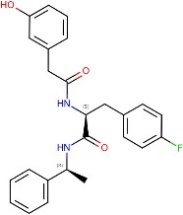 | -9.9 | 6.7221 | 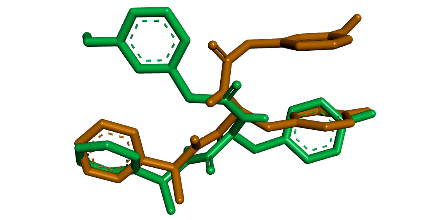 |
| 1UHL | 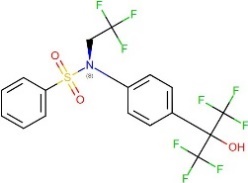 | -10.8 | 2.2775 | 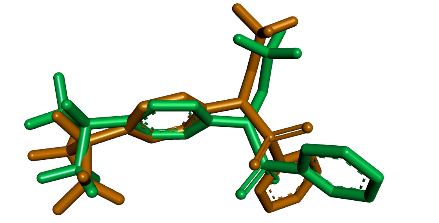 |
| 7BR3 | 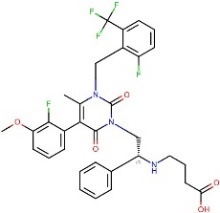 | -11.1 | 1.0019 | 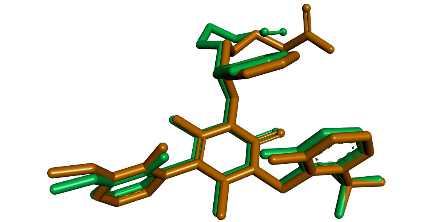 |
| BE: binding energy in kcal/mol; RMSD: root mean square distance. | | | | |

Table S2. Comparison of the initial body weight (A) and body weight gain (B) in the four experimental rat groups. Group I (Control): no exposure/treatment. Group II: *C. comosum* extract (100 mg/kg body weight/day, by gavage, for 35 days). Group III: PGB (200 mg/kg body weight/day) in drinking water for 35 days.

| Rat  groups | Group 1  (Control) | Group 2  (*C. comosum)* | Group 3  (PGB) | Group 4  (PGB + *C. comosum*) |
| --- | --- | --- | --- | --- |
| Parameters | Drinking water | *C. comosum* by gavage 100 mg/kg | PGB in drinking water for 35 days | *C. comosum* after PGB |
| Initial body weight (g) | 197.0± 11.9 | 193.8± 24.7 | 190.6±23.0 | 205.2± 26.4 |
| Body weight gain (g/d) | 4.6± 8.3 | 8.3± 0.8 | -0.2 ± 0.6^*^ | 2.51±0.7^c^ |

Data are expressed as mean ± standard deviation (SD), with n = 5 rats per group. *p < 0.05 vs. Group I; ^c^p < 0.001 vs. Group III.

Table S3. Comparison of relative liver, kidney and testis weight in the four experimental rat groups. Group I (Control): no exposure/treatment. Group II: *C. comosum* extract (100 mg/kg body weight/day, by gavage, for 35 days). Group III: PGB (200 mg/kg body weight/day) in drinking water for 35 days.

| Rat  groups | Group 1  (Control) | Group 2  *(C. comosum)* | Group 3  *(*PGB) | Group 4  (PGB *+ C. comosum*) |
| --- | --- | --- | --- | --- |
| Parameters | Drinking water | *C. comosum* by gavage 100 mg/kg | PGB in drinking water | *C. comosum* after PGB |
| Relative liver weight (g/100 g body weight) | 0.60± 0.01 | 0.61± 0.01 | 0.69± 0.01^***^ | 0.66± 0.01^a^ |
| Relative kidney weight (g/100 g body weight) | 2.65±0.055 | 2.66±0.07 | 2.91 ±0.11^*^ | 2.90±0.05^a^ |
| Relative testis weight (g/100 g body weight) | 2.03±0.02 | 2.10 ±0.01 | 2.02±0.03^**^ | 2.12±0.03^b^ |

Data are expressed as mean ± standard deviation (SD), with n = 5 rats per group. *p < 0.05, **p < 0.01, ***p < 0.001 vs. Group I; ^a^p < 0.05, ^b^p < 0.01 vs. Group III.

Table S4. Comparison of serum biomarker levels in the four experimental rat groups. Group I (Control): no exposure/treatment. Group II*: C. comosum* extract (100 mg/kg body weight/day, by gavage, for 35 days). Group III (PGB): PGB (200 mg/kg body weight/day) in drinking water for 35 days.

| Rat Groups | Triglycerides (g/l) | Cholesterol (mg/l) | Glucose (g/I) |
| --- | --- | --- | --- |
| Group 1 (Control) | 0.40±0.01 | 0.47± 0.01 | 0.97± 0.01 |
| Group 2 (*C. comosum)* | 0.30±0.01 | 0.48± 0.02 | 1.06± 0.01 |
| Group 3 (PGB) | 0.42± 0.01^*^ | 0.50± 0.02^*^ | 1.12± 0.04^***^ |
| Group 4 (PGB + *C. comosum*) | 0.40± 0.02^a^ | 0.41±0.01^b^ | 1.01± 0.05^c^ |

Data are expressed as mean ± standard deviation (SD), with n = 5 rats per group. *p < 0.05, ***p < 0.001 vs. Group I; ^a^p < 0.05, ^b^p < 0.01, ^c^p < 0.001 vs. Group III.

Table S5. Comparison of kidney function biomarker levels in the four experimental rat groups. Group I (Control): no exposure/treatment. Group II: *C. comosum* extract (100 mg/kg body weight/day, by gavage, for 35 days). Group III: PGB (200 mg/kg body weight/day) in drinking water for 35 days.

| Rat groups | Serum urea (g/l) | Serum creatinine (mg/l) | Serum uric acid (mg/l) |
| --- | --- | --- | --- |
| Group 1 (Control) | 0.51±0.01 | 5.2±0.04 | 12.46±0.80 |
| Group 2 (*C. comosum*) | 0.48±0.01 | 5.28±0.05 | 11.92±0.50 |
| Group 3 (PGB) | 0.55±0.01^***^ | 5.43±0.18^**^ | 14.68±0.90^*^ |
| Group 4 (PGB + *C. comosum*) | 0.51±0.01^b^ | 5.19±0.18^b^ | 10.28±0.70^c^ |

Data are expressed as mean ± standard deviation (SD), with n = 5 rats per group. *p < 0.05, **p < 0.01, ***p < 0.001 vs. Group I; ^b^p < 0.01, ^c^p < 0.001 vs. Group III.

Table S6. Comparison of liver function biomarker levels in the four experimental rat groups. Group I (Control): no exposure/treatment. Group II: *C. comosum* extract (100 mg/kg body weight/day, by gavage, for 35 days). Group III: PGB (200 mg/kg body weight/day) in drinking water for 35 days.

| Rat groups | AST (U/l) | ALT (U/I) | LDH (UI/I) |
| --- | --- | --- | --- |
| Group 1 (Control) | 112.6±0.8 | 40.6±44.2 | 83.6±4.7 |
| Group 2 (*C. comosum)* | 106.3±4.9 | 44.2±2.6 | 116.6±10.2 |
| Group 3 (PGB) | 131.6±5.4^***^ | 45.2±1.1** | 289.7±13.0^***^ |
| Group 4 (PGB + *C. comosum*) | 107.3±0.6^c^ | 33.8±1.7^c^ | 250.0±16.0^a^ |

Data are expressed as mean ± standard deviation (SD), with n = 5 rats per group. **p < 0.01, ***p < 0.001 vs. Group I; ^a^ p < 0.05, ^c^ p < 0.001 vs. Group III

Table S7. Comparison of hematological parameters in the four experimental rat groups. Group I (Control): no exposure/treatment. Group II: *C. comosum* extract (100 mg/kg body weight/day, by gavage, for 35 days). Group III: addition of PGB (200 mg/kg body weight/day) in drinking water for 35 days.

| Rat groups | RBC (10^6^/µL) | WBC (10^3^/µL) | PLT (10^3^/µL) | HCT (%) | Hb (g/dL) |
| --- | --- | --- | --- | --- | --- |
| Group 1 (Control) | 8.41± 0.05 | 9.60±0.50 | 786.0±20.7 | 43.0±0.2 | 15.7±0.1 |
| Group 2 (*C. comosum)* | 8.46±0.11 | 10.20±0.70 | 752.2±7.7 | 43.5±0.6 | 16.0±0.1 |
| Group 3 (PGB) | 8.37± 0.09^*^ | 7.00±0.40^**^ | 673.6±11.0^***^ | 42.0±0.5^*^ | 15.2±0.1^*^ |
| Group 4 (PGB + *C. comosum*) | 8.48±0.10^b^ | 8.90±0.40^c^ | 712.0±6.7^c^ | 43.2±0.4^c^ | 15.7±0.1^b^ |

Data are expressed as mean ± standard deviation (SD), with n = 5 rats per group. *p < 0.05, **p < 0.01, ***p < 0.001 vs. Group I; ^b^p < 0.01, ^c^p < 0.001 vs. Group III.

Table S8. Comparison of testis function biomarkers in the four experimental rat groups. Group I (Control): no exposure/treatment. Group II: *C. comosum* extract (100 mg/kg body weight/day, by gavage, for 35 days). Group III: PGB (200 mg/kg body weight/day) in drinking water for 35 days.

| Rat groups | Testosterone (nmol/l) | FSH (mIU/mL) | LH (mIU/mL) |
| --- | --- | --- | --- |
| Group 1 (Control) | 7.3±1.3 | 0.167±0.01 | 0.62±0.03 |
| Group 2 (*C. comosum)* | 6.3±0.9 | 0.17±0.01 | 0.6±0.01 |
| Group 3 (PGB) | 4.8±0.8^*^ | 0.161±0.01^**^ | 0.611±0.02^*^ |
| Group 4 (PGB + *C. comosum*) | 5.8±0.4^b^ | 0.162±0.00289^b^ | 0.62±0.23^a^ |

Data are presented as mean ± standard deviation (SD), n = 5 rats per group. *p < 0.05, **p < 0.01 vs. Group I; ^a^p < 0.05, ^b^p < 0.01 vs. Group III.

Table S9. Quantification of the histopathological changes in rat liver sections from the different experimental groups. Group I (Control): no exposure/treatment. Group II: *C. comosum* extract (100 mg/kg body weight/day, by gavage, for 35 days). Group III: PGB (200 mg/kg body weight/day) in drinking water for 35 days.

| Rat groups | Group 1 | Group 2 | Group 3 | Group 4 |
| --- | --- | --- | --- | --- |
| Parameters | Control | *C. comosum* | PGB | (PGB + *C. comosum)* |
| Inflammatory cell infiltration | - | - | +++ | - |
| Congestion | - | - | +++ | + |
| Necrosis | - | - | ++ | - |

Table S10. Quantification of the histopathological changes in rat kidney sections from the different experimental groups. Group I (Control): no exposure/treatment. Group II: *C. comosum* extract (100 mg/kg body weight/day, by gavage, for 35 days). Group III: PGB (200 mg/kg body weight/day) in drinking water for 35 days.

| Rat groups | Group 1 | Group 2 | Group 3 | Group 4 |
| --- | --- | --- | --- | --- |
| Parameters | Control | *C. comosum* | PGB | (PGB + *C. comosum)* |
| Inflammatory cell infiltration | - | - | +++ | - |
| Tubular dilation | - | - | +++ | + |
| Destroyed glomeruli | - | - | +++ | - |
| Necrosis | - | - | +++ | - |

Table S11. Quantification of the histopathological changes in rat testis sections from the different experimental groups. Group I (Control): no exposure/treatment. Group II: *C. comosum* extract (100 mg/kg body weight/day, by gavage, for 35 days).

| Rat groups | Group 1 | Group 2 | Group 3 | Group 4 |
| --- | --- | --- | --- | --- |
| Parameters | Control | *C. comosum* | PGB | (PGB + *C. comosum)* |
| Inflammatory cell infiltration | - | +++ | - | + |
| Destruction of seminiferous tubules | - | +++ | - | - |
| Destruction of germ cells | - | +++ | - | + |
| Testis necrosis | - | ++++ | - | - |
